# Does Inattention and Hyperactivity Moderate the Relation Between Speed of Processing and Language Skills?

**DOI:** 10.1111/cdev.13220

**Published:** 2019-02-09

**Authors:** Debbie Gooch, Claire Sears, Harriet Maydew, George Vamvakas, Courtenay F. Norbury

**Affiliations:** ^1^ University of Surrey; ^2^ University College London; ^3^ Royal Holloway University of London; ^4^ King's College London

## Abstract

The causal role of speed of processing (SOP) in developmental language disorder (DLD) is unclear given that SOP has been implicated in other neurodevelopmental disorders such as attention‐deficit/hyperactivity disorder. This study investigated associations between SOP, language, and inattention/hyperactivity in a U.K. epidemiological cohort (*N* = 528). Monolingual children from a range of socioeconomic backgrounds were assessed longitudinally; at ages 5–6 (2012/2013) and 7–8 years (2014/2015). Persistent weaknesses in SOP characterized children with DLD but did not predict language longitudinally. Ratings of inattention/hyperactivity moderated the association between SOP and language, indicating that SOP deficits are particularly detrimental for language when coupled with poor attention/hyperactivity. SOP may be a shared risk factor for DLD and inattention/hyperactivity or a general marker of neurodevelopmental disorder.

Developmental language disorder (DLD) is a common disorder affecting ~10% of children during the first year of school (Norbury, Gooch, Wray, et al., 2016). DLD is the term recently adopted by an international consortium of experts; it includes children previously identified as having “specific language impairment (SLI)” and will be used throughout this article for consistency (Bishop et al., 2017). DLD is characterized by difficulties in the use of language structure (e.g., phonology, semantics, syntax, and morphology) and the effective use of language in different social contexts (pragmatics). Approximately 7.5% of children have DLD that cannot be explained by factors such as sensory loss, neurological damage or known medical diagnoses, for example, autism/intellectual impairment, whereas an additional 2.3% have language disorder that is associated with another known medical diagnosis (Norbury, Gooch, Wray, et al., 2016). This is broadly in line with previous epidemiological estimates of “SLI” at school entry (12.5% Beitchman, Nair, Clegg, & Patel, 1986 and 7.4% Tomblin et al., 1997). DLD persists into adolescence and is associated with poor academic, employment, and social outcomes (Johnson et al., 1999). Research has therefore aimed to identify central causal mechanisms of DLD that may help to inform early clinical diagnosis and appropriate interventions (Conti‐Ramsden, Botting, & Faragher, 2001). In light of this, this study aims to consider the potential causal role of general speed of processing (SOP) deficits in DLD by assessing these skills longitudinally in an epidemiological cohort of children (aged 5–6 years and 7–8 years old). Furthermore, a novel aspect of this study is to investigate whether individual differences in symptoms of inattention/hyperactivity act to moderate any relation between SOP and language.

## SOP in DLD

An influential theory, “The Generalized Slowing Hypothesis,” posits that DLD is caused by a domain general processing dysfunction that results in proportionally slower SOP across all aspects of mental processing (Kail, 1994; Lahey, Edwards, & Munson, 2001; Miller, Kail, Leonard, & Tomblin, 2001). SOP refers to the efficiency of the cognitive processes required to comprehend and act upon stimuli in order to complete a task (Shanahan et al., 2006); input must be processed before information decays or is disrupted by other interfering information. Language development is thought to be particularly vulnerable to this “generalized slowing” of cognitive processes given the time‐dependent nature of speech processing (e.g., identifying linguistically relevant information from a speech stream or processing particular grammatical morphemes) compared to that of other cognitive processes (Miller et al., 2001).

Numerous empirical studies report weaknesses in SOP in individuals with DLD relative to typically developing (TD) peers across both linguistic and nonlinguistic tasks, lending support to the generalized slowing hypothesis. Children with DLD are slower than age‐matched peers on perceptual motor tasks (Powell & Bishop, 1992), nonlinguistic cognitive tasks (e.g., mental rotation and visual discrimination; Schul, Stiles, Wulfeck, & Townsend, 2004), and speeded language tasks (e.g., word‐recognition; Montgomery & Leonard, 1998). Furthermore, performance on SOP tasks is moderately predictive of DLD status (Park, Mainela‐Arnold, & Miller, 2015).

The theory, however, remains controversial with conflicting findings across studies. The statistical methods used in some meta‐analytic studies have been challenged (Windsor, Milbrath, Carney, & Rakowski, 2001), and re‐analysis of the data suggest that, given the large variability in findings between and within studies, the overall differences in SOP between children with and without DLD are not statistically significant. In addition, weaknesses in SOP appear neither necessary nor sufficient to result in DLD. Not all children with DLD show poor SOP, with only 37.5%–75% of children with DLD presenting with slower SOP compared to age‐matched peers (Miller et al., 2001). The fact that poor SOP is apparent in children with attention‐deficit/hyperactivity disorder (ADHD) who have verbal skills within the normal range (Oram Cardy, Tannock, Johnson, & Johnson, 2010; Shanahan et al., 2006) further calls into question the causal role this cognitive impairment plays in DLD. In addition, findings regarding the relation between individual differences in SOP and language skill are inconsistent with one study finding no direct linear correlations between SOP and standardized language test scores (Lahey et al., 2001), whereas another, which used a latent variable approach, found that a general SOP factor predicted small (17%) but significant unique variance in individual difference in language skill (Leonard et al., 2007). To date, few studies have investigated the longitudinal relations between SOP and language although there is initial evidence to suggest that early SOP (age 3 years) is directly related to later lexical skills (receptive vocabulary and verbal fluency at 13 years) once early lexical skills were controlled (Rose, Feldman, & Jankowski, 2015). Finally, given that SOP tasks often require higher level processes such as attention for their successful execution, it is possible that weaknesses in SOP in DLD reflect comorbidity rather than a putative cause of DLD (Oram Cardy et al., 2010). Related to this, there is a parallel line of research that considers poor SOP to be a shared cognitive risk factor for ADHD and reading disorder (RD; Pennington, 2006), both of which frequently co‐occur with DLD. This view largely comes from studies demonstrating that poor SOP is related to cases of RD and ADHD but that these deficits are underadditive in children with comorbid ADHD + RD (Shanahan et al., 2006), as well as evidence that individual variation in SOP predicts both individual variation in reading skills and inattention (McGrath et al., 2011).

## SOP in Relation Inattention/Hyperactivity

A number of studies have highlighted associations between inattention/hyperactivity and SOP in children with ADHD. ADHD is a commonly occurring developmental disorder characterized by symptoms of inattention, hyperactivity, and impulsivity and has a reported school‐aged prevalence of 5%–7% (Willcutt, 2012). Like DLD, ADHD, and symptoms of inattention/hyperactivity have been associated with slower (and more variable) SOP (Gooch, Snowling, & Hulme, 2012; Kuntsi & Stevenson, 2000; Scheres, Oosterlaan, & Sergeant, 2001) and impairments in processing efficiency have been implicated in theoretical models of ADHD (e.g., Sergeant, 2005). ADHD frequently co‐occurs with DLD (Cohen et al., 2000; Mueller & Tomblin, 2012; Sciberras et al., 2014) and thus it is necessary to establish whether weaknesses in SOP are uniquely associated with DLD and poor language skills, whether they covary with symptoms of ADHD (i.e., increased inattention/hyperactivity) or whether they reflect a risk factor for comorbidity and/or neurodevelopmental disorder more generally. To date only one study has examined SOP in children with DLD compared to those with ADHD (Oram Cardy et al., 2010), although a comorbid ADHD+DLD comparison group was not included. Oram Cardy et al. (2010) found that children with ADHD had slower SOP relative to both children with DLD and age‐matched TD peers. Children with DLD also had slower SOP than TD children, but it was those with ADHD who showed the greatest impairment. These findings challenge the hypothesis that SOP deficits cause DLD. Rather, poor SOP may be primarily related to symptoms of ADHD or indeed a putative shared risk factor for disorders of language learning and attention (Pennington, 2006).

## The Current Study

To date, numerous tasks have been used to measure SOP, and we do not know whether they all tap the same underlying cognitive construct. Performance on speeded tasks are likely influenced by the speed at which individuals perceive and process incoming information, as well as the speed at which they are able to make their response. Indeed, studies have suggested that it is output speed rather than input or perceptual speed that is impaired in ADHD (Sergeant, 2005). In the absence of a clearly specified theory of SOP, we used the working definition suggested by Shanahan et al. (2006): SOP is the underlying cognitive efficiency of understanding and acting upon external stimuli, which includes integrating low‐level perceptual speed, higher level cognitive speed, and output speed. We therefore included several measures of SOP that require children to rapidly process input, make a decision, and then respond to different types of information. Although the tasks tap different aspects of higher order cognition, they all also tap the more general construct of processing speed.

Previous research has demonstrated concurrent weakness in SOP in relatively small groups of children clinically referred for DLD compared to age matched TD peers. A novel feature of this study is that we assessed SOP in a population cohort with a wide range of language skills, thus our comparison of children with and without DLD is free from Berkson's bias (i.e., referral bias resulting in children with more complex/co‐occurring conditions being more likely to be referred and thus altering the composition of clinically ascertained samples; Mueller & Tomblin, 2012). Furthermore, we treated our data continuously to explore the relations between individual differences in SOP, children's language skills, and ratings of inattention/hyperactivity. This approach avoids the limitations of cut offs for diagnostic categories that are relatively arbitrary and more likely reflect a continuum of psychological skill (Frith, 2001). In addition, data from two time points (5–6 years old and 7–8 years old) enable us to explore the longitudinal relations between SOP, children's later language and symptoms of inattention/hyperactivity.

This study specifically aimed to first establish whether children with DLD have weaknesses in SOP compared to children without DLD as predicted by the generalized slowing hypothesis. Second, we used latent variables to investigate how the common underlying cognitive processes involved in SOP tasks are related to individual differences in language skill and how variations in symptoms of inattention/hyperactivity moderate this relation. Finally, we used a cross‐lagged model to establish whether SOP longitudinally predicts individual variation in core language skills and/or symptoms of inattention/hyperactivity.

On the basis of previous research, we predicted that children with DLD would have slower SOP compared to children without DLD. We also predicted that children with DLD would have more symptoms of inattention/hyperactivity (using ratings from the Strengths and Weaknesses of ADHD symptoms and Normal behavior scale [SWAN]) compared to children without DLD, given the frequent comorbidity between DLD and ADHD reported in the literature (Cohen et al., 2000; Mueller & Tomblin, 2012; Sciberras et al., 2014). Finally, we asked whether the relation between SOP and children's language skills would be moderated by their inattention/hyperactivity. Given that the generalized slowing hypothesis suggests that weakness in SOP play a causal role in DLD, we predicted that early individual variation in SOP would be more strongly related to later individual variation in language skills than vice versa.

## Method

### Study Population

Participants are from the Surrey Communication and Language in Education Study (SCALES), a longitudinal population study of DLD at school entry (Norbury, Gooch, Wray, et al., 2016). In Stage 1, teachers in state‐maintained schools in Surrey, England completed an online questionnaire that included an assessment of language proficiency, the Children's Communication Checklist‐Short (CCC‐S; see Norbury, Gooch, Baird, et al., 2016) for 7,267 children who had started school in September 2011. All children were aged between 4 years 9 months and 5 years 10 months at the time of assessment, which took place in the summer (third) term of the first year of school (i.e., reception year in the United Kingdom, which is equivalent to kindergarten in the United States). Income Deprivation Affecting Children Index (IDACI) scores obtained from home postcodes provided a measure of socioeconomic status (SES; McLennan, Barnes, Davies, Garratt, & Dibben, 2011). Index scores in England range from 1 (most deprived) to 32,844 (*M* = 16,241), and in this sample ranged from 731 (most deprived) to 32,474 (most affluent; *M* = 21,592, *SD* = 7,830). There were 51.11% boys, and 82.28% of the screened sample were of White ethnic origin, consistent with recent census data for Surrey and the United Kingdom.

In Stage 2, a subsample of children assessed at Stage 1 was selected for in‐depth assessment in Year 1 (ages 5–6 years) and Year 3 (ages 7–8 years) using stratified random sampling. Initial strata identified children who were reported as having “no phrase speech” (NPS; i.e., reported expressive language level of two‐word utterances or less; *n* = 89), those attending special schools for severe learning disabilities (*n* = 31) and those for whom English was an additional language (*n* = 777). Children in special schools were excluded from further study due to staff concerns about their ability to participate in assessments. Children with English as an additional language were invited to a different study; their data are not included here. All remaining children with NPS (*n* = 48) were invited for in‐depth assessment.

For the remaining monolingual children (*n* = 6,411), cut‐off scores on the CCC‐S were derived separately for each of three age groups (autumn, spring, and summer) to identify sex specific strata of boys (13.9%) and girls (14.8%) with teacher ratings of poor language (i.e., high risk of DLD was defined as scores at or above the 86th centile for sex and age group). In total, 636 children (including 48 NPS) were invited to participate with a higher sampling fraction for high risk (40.5% for boys and 37.5% for girls) versus low risk (4.3% for boys and 4.2% for girls) children. In Year 1, 529 monolingual children (83% of invited cohort) were assessed in detail, although one child was unable to provide sufficient assessment data to allow classification and thus is not included here (50.20% male and 91.16% white ethnic origin). In Year 3, 499 monolingual children (95% of assessed cohort) were seen for follow‐up assessment (50.97% male and 90.83% white ethnic origin; see recruitment flow diagram in Figure S1 and participant descriptive statistics in Table 1).

**Table 1:** Weighted Sample Characteristics for Year 1 and 3

	Year 1		Year 3	
*N* raw (estimated)	528	(6,442.35)	499	(6,463.51)
% Male	50.2	(44.19, 56.20)	50.97	(44.81, 57.10)
% White	91.16	(87.05, 94.06)	90.83	(86.57, 93.84)
Age	71.78	(71.21, 72.35)	95.26	(94.71, 95.82)
SES (IDACI rank)	23,094.87	(22,166.46, 24,023.28)		
NVIQ (*z*‐score)	0.00	(0.06, −0.12)		
Language measures (*z*‐scores)				
EOWPVT	−0.01	(−0.13, 0.11)	0.01	(−0.10, 0.13)
ROWPVT	−0.02	(−0.14, 0.10)	0.02	(−0.10, 0.13)
Sentence repetition	−0.02	(−0.13, 0.10)	0.02	(−0.09, 0.12)
TROG‐short	−0.03	(−0.15, 0.08)	0.03	(−0.09, 0.15)
Narrative recall	0.03	(−0.15, 0.09)	0.03	(−0.09, 0.15)
Narrative comprehension	0.00	(−0.12, 0.12)	0.01	(−0.10, 0.13)
Speed of processing measures				
Coding/65	35.03	(33.54, 36.52)	48.61	(47.32, 49.90)
Visual search rate	0.23	(0.22, 0.24)	0.30	(0.29, 0.31)
RAN rate	0.88	(0.84, 0.91)	0.88	(0.85, 0.92)
Simple Reaction Time: mean RT (s)	482.77	(473.00, 492.53)	404.43	(396.93, 411.93)
SWAN ratings (teachers)				
Inattention/63^b^	38.91	(37.07, 40.75)	40.52	(38.81, 24.24)
Hyperactivity/63^b^	41.96	(40.24, 43.67)	42.95	(41.36, 44.53)
Total/126^b^	80.86	(77.49, 84.24)	83.47	(80.29, 86.65)

Note

Estimated means and frequencies reported, with 95% CIs in parentheses. SES = socioeconomic status; IDACI = Income Deprivation Affecting Children Index; EOWPVT = Expressive One Word Picture Vocabulary Test; ROWPVT = Receptive One Word Picture Vocabulary Test; TROG = Test for Reception of Grammar; SRT = Simple Reaction Time, RT = reaction time; RAN = rapid automatized naming; SWAN = Strengths and Weaknesses of Attention‐Deficit/Hyperactivity Disorder Symptoms and Normal Behavior scale.

^a^Year 1: *N* = 343 (6,394.08), Year 3: *N* = 362 (6,396.65).

### Consent Procedures

Consent procedures and study protocol were developed in consultation with Surrey County Council and approved by the Research Ethics Committee at Royal Holloway, University of London, where the study originated. Opt‐out consent was adopted for Stage 1 as data could be provided anonymously to the research team; 20 families opted out. For Stage 2, written, informed consent for two episodes of direct assessment was obtained from the parents or legal guardians of all participants. Verbal assent was obtained from the children themselves. Prior to assessment in Year 3, families received an additional information sheet and could at this point withdraw from the study; 18 families withdrew consent, 5 moved abroad, 3 could not be contacted, and 3 provided insufficient data on the day of testing for diagnostic classification. Of the 29 children (19 male) not included in follow‐up, 22 had been classified as “TD” in Year 1 and had no evidence of language, learning, or behavioral difficulties.

### Assessment Procedures

In‐depth assessments were conducted at the child's school by a trained SCALES researcher and lasted approximately 2 hr (with breaks). The same tasks/rating scales were used to assess language, SOP, and inattention/hyperactivity at both time points (see Table S2 for psychometric estimates for each measure where available). Testers were given a suggested order in which to administer tasks, but this was not fixed.

### Language

The assessment of children's language skills closely followed procedures which have informed *Diagnostic and Statistical Manual of Mental Disorders*, 5th ed. (*DSM–V*) criteria for DLD. Six language tasks measured expressive and receptive language skills across multiple domains (Tomblin et al., 1997): vocabulary (*Expressive/Receptive One Word Picture Vocabulary Test, 4th ed*.; Martin & Brownell, 2010), grammar (a short form of the *Test for Reception of Grammar, 2nd ed*. (Bishop, 2003) and the *School‐Age Sentence Imitation Test‐E32* (Marinis, Chiat, Armon‐Lotem, Piper, & Roy, 2011), and narrative (narrative recall and comprehension adapted from the *Assessment of Comprehension and Expression 6–11*; Adams, Cooke, Crutchley, Hesketh, & Reeves, 2001). In Year 1, DLD was defined as scores of −1.5 *SD* or below on two of the five resulting language composites (expressive, receptive, vocabulary, grammar, and narrative; see Supporting Information for task descriptions and details). As explained below, our sample of children with DLD included those with idiopathic DLD (more akin to SLI) as well as those with DLD associated with a known medical diagnosis/intellectual impairment (Norbury, Gooch, Wray, et al., 2016).

### Nonverbal IQ and Clinical Diagnosis

Nonverbal IQ (NVIQ) was estimated using a composite of *block design* and *matrix reasoning* scores from the Wechsler Preschool and Primary Scale of Intelligence, 3rd ed. (WPPSI–III U.K.; Wechsler, 2003) at first assessment and the Wechsler Intelligence Scales for Children, 4th ed. (U.K.; Wechsler, 2004) at follow‐up. Information regarding any known medical diagnoses was elicited from teachers during Stage 1 and from parents and/or the school special educational needs co‐ordinator during the Stage 2 assessments. One of the key strengths of the current sample is that it is a population sample, and thus children with intellectual disability (Nonverbal IQ composite more than 2 *SD* below the population mean) and/or existing medical diagnoses (e.g., Autism Spectrum Disorders, genetic/neurological disorders, visual/hearing impairment; *N* = 61) have not been excluded from the main analyses (see Table S1 for a breakdown of the diagnoses in this group). Indeed, covarying nonverbal IQ is inappropriate in this case as it is not nonrandomly associated with group membership (Dennis et al., 2009). Instead, to facilitate comparisons with the previous literature that use criteria for “specific” language impairment, parallel analyses excluding the 61 children with known medical diagnoses and/or intellectual disability are reported in Supporting Information (key findings are also presented in the figures); these analyses yield the same pattern of results.

### Speed of Processing

#### Rapid Automatized Naming

Children were asked to name 40 familiar objects (dog, eye, key, lion, table) as quickly as possible (Hulme, Nash, Gooch, Lervag, & Snowling, 2015). All children were able to correctly name each of the objects on a practice trial. The time taken and number of errors were recorded and a rapid automatized naming (RAN) rate score (time taken/items correct) was calculated.

#### Visual Search (Apples Task)

Children were asked to search an array comprising targets (18 red apples) and distractors (81 red strawberries, 81 white apples; Breckenridge, 2008). The child was given 1 min to find as many targets as they could. Hits (number of targets correctly identified) and commission errors (identifying a distractor) were recorded. A visual search efficiency score was calculated (hits—commission errors/60 s), with a high score indicating more efficient SOP.

#### Coding (WPPSI–III)

Following an example, children were asked to copy symbols into simple geometric shapes (Wechsler, 2003). After five practice items, each child was given 120 s to draw symbols into 59 shapes. The child was instructed to work in order from left to right, top to bottom. The child scored one point for every shape they successfully completed and were given bonus points if they accurately completed the task under the time limit (max score 65).

#### Simple Reaction Time

In this computerized task, the child was instructed to press a response key as quickly as possible when a stimulus (bug) appeared. The stimulus was preceded by a fixation cross and a varied lag (300, 600, or 900 ms): Children had 2,000 ms from the onset of the stimulus to make their response. Children completed three practice trials followed by 30 test trials. Mean reaction time (RT) was calculated for each child. Errors of omission were low (7% of the total trials), thus each child contributed between 20 and 30 RTs (average 27 trials) to the calculation of RT means. Furthermore, data have not been trimmed to remove outlying values (Ulrich & Miller, 1994); however, given the nondecision portion of simple RT is approximately 100 ms (Luce, 1986), all RTs of < 100 ms (<3% of the total trials) were discarded as anticipatory errors.

### Inattention/Hyperactivity

To assess inattention/hyperactivity, the SWAN (Swanson et al., 2012) was completed by 344/529 teachers (35% missing) in Year 1 and 362/499 (27% missing) in Year 3. There were no significant differences between those who had questionnaires returned and those who did not in age, SES (IDACI scores), percent male, or teacher ratings of language and communication skills (CCC‐S), behavior (SDQ; Goodman, 1997), and early educational attainment (Early Years Foundation Stage Profile: Department of Education, 2013) taken at Stage 1. SWANs were also completed by parents 299/529 parents (43% missing) in Year 1 and 282/499 (43% missing) in Year 3. Given that fewer completed SWAN were available from parents and that there was evidence of nonresponse bias (i.e., children for whom parental questionnaires were not returned had lower language skills, lower educational attainment and were from lower SES backgrounds compared to those whose parents returned questionnaires), the main analyses here were conducted using only teacher ratings. Analyses of parent SWANs (confirmatory factor analysis (CFA) and moderation analysis) are presented in Supporting Information.

The SWAN measures behaviors associated with ADHD (inattention, hyperactivity, and impulsivity) based on *DSM–IV* (American Psychiatric Association, 2013) criteria. It includes 18 positively phrased items; nine items tapping symptoms of Inattention (e.g., Give close attention to detail and avoid making mistakes); and nine tapping symptoms of Hyperactivity/Impulsivity (e.g., Awaits turn/stands in line and take turns). The SWAN is dimensional at the item level to overcome issues that characterizes many other ADHD rating scales (Swanson et al., 2012), namely that scores are not normally distributed in the population as items focus on the presence of difficulty. Thus the SWAN captures variance at both the positive and negative ends of the symptom dimension (Arnett et al., 2013).

For each item respondents were asked to compare the child's attention/behavioral skills to those of his/her peers using a 7‐point Likert scale (*far below average* = 1, *below average* = 2, *somewhat below average* = 3, *average* = 4, *somewhat above average* = 5, *above average* = 6, and *far above average* = 7; e.g., Polderman et al., 2007). The maximum score on the SWAN is 126 (63 for each of the subscales: Inattention and Hyperactivity/Impulsivity). A low score reflects more evidence of inattention/hyperactivity. Cronbach's alphas in the current sample indicated good internal reliability for the SWAN and its subscales as completed by teachers (*r*'s = .94–.98).

It is important to recognize that the SWAN measures relative strengths and deficits in attention/hyperactivity; it is not a stand‐alone diagnostic instrument. In this study, we were interested in investigating the full range of attention profiles, not just the impact of co‐morbid ADHD. Although it is possible to identify possible “cases” using SWAN algorithms, these should be treated with utmost caution. Indicative cases are reported in Supporting Information and discussed in detail elsewhere (Gooch, Maydew, Sears, & Norbury, 2017).

### Sampling Weights and Missing Data

Sampling weights were constructed as the inverse of the predicted probability of a child being included in the study, so that when weighted, the estimates obtained from the sample are estimates for the whole population. Separate weights were constructed for analyses that utilize language and SOP measures alone and for analyses that include SWAN responses as well. This was due to the different proportions of missing data between the different types of outcome variables. Predicted probabilities of inclusion were estimated via two logistic models; the first logistic model was fitted to the entire population of 6,459 children recruited during Phase 1. The model estimated the probability of a child's participation in Phase 2 and included covariates predictive of inclusion due to study design. These were total number of pupils assessed per school and whether the child was identified as having high risk of DLD based on CCC‐S teacher ratings (86th centile or above for sex and age group). The second logistic model was fitted only to children selected for the second phase of the study. For analyses that utilized language and SOP measures, complete data existed on 528/636 children for Year 1 and on 499/528 for Year 3. For analyses that utilized SWAN responses too, complete data existed for 343/528 children at first assessment and 362/499 children at second assessment. The models estimated the probability of a child's inclusion in Stage 2 due to individual characteristics of the participants. All covariates predictive of “missingness” such as sex, season of birth, IDACI rank score, learning English as an additional language, CCC‐S total raw score, SDQ total difficulties score, and school level factors such number of pupils on role, percentage girls, percentage with identified special education needs, and percentage receiving free school meals (a measure of school‐level deprivation) were tested in a stepwise elimination process and included in the model (at a cut‐off point of .2) in order to maximize the likelihood of the data being missing at random. The final weights were a multiplication of the inverse of the predicted probabilities from the two models.

The weighted estimates are thus estimates for the whole U.K. mainstream school population (excluding those with English as an additional language). Weighted descriptives for the sample on all measures are shown in Table 1 (unweighted descriptives are shown in Table S3).

### Statistical Analysis

Language and SOP raw scores were standardized using the LMS method (Cole & Green, 1992). LMS is a method of standardization, which allows for skewed measurements and was used for the construction of test norms for SCALES (Vamvakas, Norbury, Vitoratou, Gooch, & Pickles, 2017). The resulting scores were estimated by utilizing the weights we produced and reflect standardized scores adjusted for age with a mean of 0 and a standard deviation of 1. These standardized scores were then used in all subsequent statistical analyses.

Statistical analyses were conducted using Stata's (v14) suite of survey data commands *svy* (Stata Corporation, 2015). Structural equation modeling (SEM) with latent variables was used to investigate whether SOP was related to children's language skills concurrently and longitudinally, and whether individual differences in inattention/hyperactivity moderated this relation across the entire population sample. All SEM analyses were weighted and run in M‐Plus (Muthen & Muthen, 1998) under the robust maximum likelihood estimator, which uses a pseudo‐maximum likelihood asymptotic covariance matrix (Asparouhov, 2005).

Given that chi‐squared values are affected by sample size (Bollen, 1989), we used the following general guidelines for reasonable model fit: comparative fit index (CFI) and Tucker–Lewis index (TLI) close to .95 (and > .90) in combination with a cut‐off value close to .09 for standardized root mean square residual (SRMR) or root mean square error of approximation (RMSEA) close to .05 and SRMR > .06 (Hu & Bentler, 1999). Fit statistics for moderation analyses have not yet been developed, thus we provide information regarding model fit for the measurement model that formed the basis of this analysis.

For the main longitudinal model, measurement invariance was established across the two time‐points using STATA v15, which allows for adjusted Wald tests comparisons for the weighted models. Three forms of invariance were examined: metric, scalar, and strict. For metric invariance, loadings of each factor were constrained to be equal across time points and conducted joint Wald tests. The Wald *p*‐value from the joint comparison of the factor loadings for Language at Year 1 and Year 3 was .4898, the corresponding *p*‐values for SOP and inattention/hyperactivity were .1074 and .3963, respectively. For scalar invariance, first factor loadings were constrained to be equal and then equality of intercepts was assessed. The Wald *p*‐value from the joint comparison of the intercepts of Language at Year 1 and Year 3 was .7693; the corresponding *p*‐value for SOP and inattention/hyperactivity were .9524 and .0528, respectively. Finally, to check strict invariance, both factor loadings and intercepts were constrained to be equal and invariance of the residual variances was examined. The Wald *p*‐value from the joint comparison between the residual variances of the Language items at Year 1 and Year 3 was .7916, the corresponding *p*‐value for the SOP items and the inattention/hyperactivity items were .1117 and .0520, respectively.

## Results

Table 2 reports descriptive statistics for both language groups on the six language measures administered in Year 1 and 3. As predicted, children with DLD also received significantly lower SWAN ratings (indicating increase levels of inattention/hyperactivity) from their teachers compared to children without DLD at both time points (a similar pattern of findings obtained when those with known clinical diagnoses were excluded from the analysis, see Table S4 and Figure S2).

**Table 2:** Participant Characteristic for Those With and Without Developmental Language Disorder (DLD) in Years 1 and 3

Year 1	TD language		DLD		*F*(1, 527)	*p*
*N* raw (estimated)	392	(5, 803)	136	(639)		
% Male^a^	49.17	(42.72, 55.64)	59.60	(44.57, 73.03)	1.60	.21
% White^a^	91.07	(86.48, 94.21)	91.96	(86.58, 95.30)	0.09	.76
Age	71.77	(71.14, 72.39)	71.89	(70.83, 72.95)	0.04	.84
IDACI rank	23,779.48	(22,833.94, 24,725.03)	16,877.60	(14,314.78, 19,440.42)	24.64	< .001
NVIQ (*z*‐score)	0.11	(−0.01, 0.23)	−1.02	(−1.24, −0.80)	76.80	< .001
Language measures (*z*‐scores)						
EOWPVT	0.14	(0.02, .26)	−1.43	(−1.57, −1.28)	274.54	< .001
ROWPVT	0.14	(0.02, 0.25)	−1.43	(−1.67, −1.18)	127.61	< .001
Sentence repetition	0.15	(0.04, 0.26)	−1.55	(−1.83, −1.26)	121.88	< .001
TROG‐short	0.11	(−0.01, 0.23)	−1.30	(−1.46, −1.13)	183.16	< .001
Narrative recall	0.11	(−0.01, 0.23)	−1.38	(−1.61, −1.15)	128.71	< .001
Narrative comprehension	0.17	(0.05, 0.28)	−1.53	(−1.80, −1.26)	131.75	< .001
SWAN ratings (teachers)						
Inattention^b^	40.78	(39.02, 42.54)	27.67	(22.56, 32.78)	22.79	< .001
Hyperactivity^b^	42.99	(41.18, 44.80)	33.67	(28.40, 38.93)	10.87	< .01
Total^b^	83.77	(80.35, 87.19)	61.33	(51.10, 71.56)	16.74	< .001

Year 3	TD language		DLD		*F*(1, 498)	*p*
*N* raw (estimated)	370	(5,808)	129	(656)		
Language measures (*z*‐scores)						
EOWPVT	0.17	(0.06, 0.29)	−1.40	(−1.61, −1.20)	168.67	< .001
ROWPVT	0.15	(0.03, 0.27)	−1.18	(−1.43, −0.94)	94.84	< .001
Sentence repetition	0.17	(0.07, 0.27)	−1.37	(−1.77, −0.97)	53.65	< .001
TROG‐short	0.18	(0.06, 0.30)	−1.29	(−1.52, −1.06)	126.38	< .001
Narrative recall	0.14	(0.01, 0.26)	−0.92	(−1.23, −0.61)	38.35	< .001
Narrative comprehension	0.15	(0.03, 0.27)	−1.20	(−1.43, −0.98)	108.88	< .001
SWAN ratings (teachers)						
Inattention^c^	42.34	(40.67, 44.01)	25.89	(21.96, 29.81)	57.50	< .001
Hyperactivity^c^	44.22	(42.56, 45.87)	32.70	(30.05, 35.35)	52.63	< .001
Total^c^	86.56	(83.37, 89.75)	58.59	(52.33, 64.84)	61.45	< .001

Note

Estimated means and frequencies reported, with 95% CIs in parentheses. TD = typically developing; IDACI = Income Deprivation Affecting Children Index; EOWPVT = Expressive One Word Picture Vocabulary Test; ROWPVT = Receptive One Word Picture Vocabulary Test; TROG = Test for Reception of Grammar; SWAN = Strengths and Weaknesses of Attention‐Deficit/Hyperactivity Disorder Symptoms and Normal Behavior scale.

^a^ *F* statistic is a designed based corrected χ^2^ value. ^b^Year 1: *N* = 343 (6,394.080). ^c^Year 3: *N* = 362 (6,396.65).

**Figure 1:**
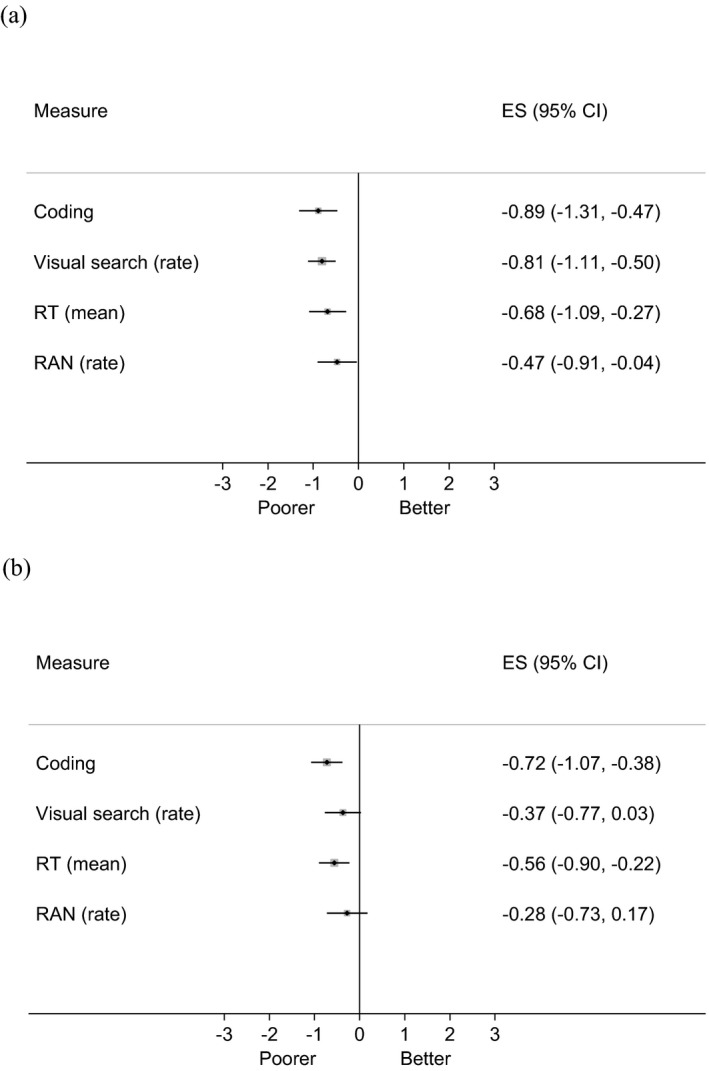
Standard *z*‐score differences between children with and without language disorder on measures of speed of processing in Year 1 (a) and Year 3 (b). Error bars are 95% confidence intervals. Bars that cross the zero midline indicate no significant group difference. Boxes to the left of the zero indicate poorer performance in the developmental language disorder group. RT = reaction time; RAN = rapid automatized naming.

Subsequent analyses compared children with and without DLD on the four individual measures of SOP at both assessment points; effect sizes and 95% CIs for these comparisons are shown in Figure 1 (effect sizes and CIs for the RT measures have been multiplied by −1 for display purposes). Within this epidemiological sample, children with DLD as a group performed significantly worse than children without DLD on all of the measures of SOP administered at 5–6 years of age: coding *F*(1, 507) = 17.07, *p *<* *.001, visual search, *F*(1, 521) = 27.03, *p *<* *.001, simple RT, *F*(1, 510) = 10.70, *p *<* *.01 and RAN, *F*(1, 506) = 4.59, *p *<* *.05. By age 7–8 years, significant differences between children with and without DLD on measures of SOP were only evident on the coding task, *F*(1, 482) = 16.90, *p *<* *.001 and simple RT, *F*(1, 488) = 10.71, *p *<* *.01.

### Does SOP Predict Children's Later Language Skills?

Latent factors represent continuously distributed symptom dimensions underlying the diagnostic category of DLD, as well as a measure comprising indices of both verbal and nonverbal SOP were created. We allowed errors to correlate for similar tasks within the language dimension (namely the two narrative tasks) to allow for task specific factors (e.g., instructions/questions and administration) and/or child factors (e.g., fatigue). Our proposed measurement models fit the data at both assessment points well (*N* = 499; weighted to give estimates for the population): CFI = .98, TLI = .97, RMSEA = .03 (95% CIs [.01, .05]), SRMR = .04 and CFI = .94, TLI = .92, RMSEA = .05 (95% CIs [.04, .07]), SRMR = .05, respectively.

**Figure 2:**
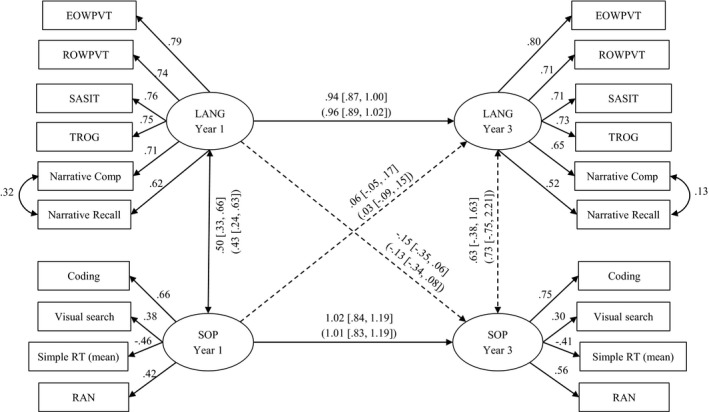
Longitudinal autoregressive path model with cross‐lagged effect showing the relation between language (LANG) and speed of processing (SOP). Standardized path estimates and correlation coefficients are depicted by single and double‐headed arrows, respectively. Path weights and confidence intervals for the whole sample are shown outside the brackets (*N* = 499), those for the sample without children with known diagnoses are shown inside the brackets (*N* = 441). Paths significant at the 0.05 level are represented by solid lines; nonsignificant paths by dashed lines. EOWPVT = Expressive One Word Picture Vocabulary Test; ROWPVT = Receptive One Word Picture Vocabulary Test; SASIT = School‐Age Sentence Imitation Test; TROG = Test for Reception of Grammar; RT = reaction time; RAN = rapid automatized naming.

To assess longitudinal relations between SOP and language, a latent variable autoregressive path model with cross‐lagged effects was fitted to the data for the whole sample. This model (see Figure 2) assesses the longitudinal stability of language skills (receptive and expressive vocabulary, grammar, and narrative) and SOP (coding, visual search, RAN, and mean RT), and also assesses whether SOP predicts additional variance in language across time. If such longitudinal cross loadings were present, they would be consistent with (but not prove) a causal influence from the earlier to the later variable.

The resulting model, shown in Figure 2, gives good fit to the data: CFI = .96, TLI = .95, RMSEA = .04 (90% CI [.03, .04]), SRMR = .05. The figure shows standardized path weights together with their 95% confidence intervals for the whole sample (estimates excluding those with known clinical diagnoses are shown in brackets). Paths significant at the .05 level are represented by solid lines; nonsignificant paths by dashed lines.

It is notable that the latent variables describing language and SOP show impressive longitudinal stability. The model accounted for 94% of the variance in language skills at age 7–8 and 91% variance in SOP at ages 7–8. In addition, all cross‐lagged effects are small, and none are close to being significant.

### Do Individual Differences in Inattention/Hyperactivity Moderate the Relation Between SOP and Language?

**Figure 3:**
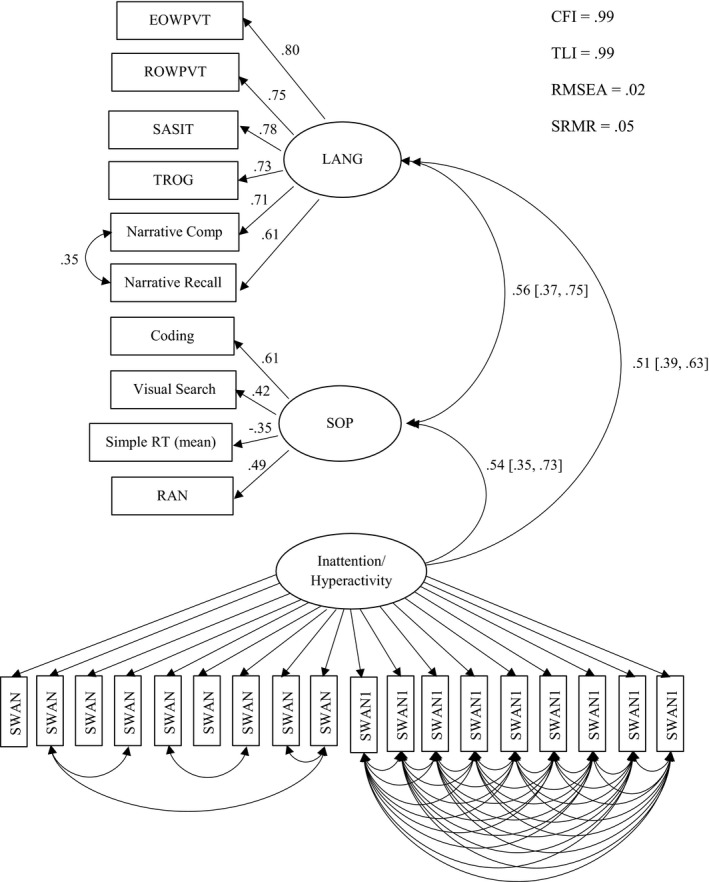
Measurement model for the continuously distributed dimensions of speed of processing, language, and inattention/hyperactivity (as measured by the Strengths and Weaknesses of Attention‐Deficit/Hyperactivity Disorder symptoms and Normal behavior scale [SWAN]) in Year 1 (*N* = 343). Standardized path estimates and correlation coefficients (with 95% CIs) are depicted by single and double‐headed arrows, respectively (see Table S5 for the SWAN latent variable path estimates: all loadings range between .72–.94). EOWPVT = Expressive One Word Picture Vocabulary Test; ROWPVT = Receptive One Word Picture Vocabulary Test; SASIT = School‐Age Sentence Imitation Test; TROG = Test for Reception of Grammar; RT = reaction time; RAN = rapid automatized naming; CFI = comparative fit index; TLI = Tucker–Lewis index; RMSEA = root mean square error of approximation; SRMR = standardized root mean square residual.

The measurement model for SOP, language, and inattention/hyperactivity at age 5–6 (weighted to give estimates for the population) is displayed in Figure 3. The language latent factor represents continuously distributed symptom dimensions underlying the diagnostic category of DLD, the inattention/hyperactivity latent factor represents individual differences in inattention/hyperactivity as measured by the SWAN, and the SOP latent factor comprises indices of both verbal and nonverbal SOP. We allowed errors to correlate for similar tasks within the language dimension (namely the two narrative tasks) to allow for task specific factors (e.g., instructions/questions and administration) and/or child factors (e.g., fatigue). We also allowed errors to correlate for the SWAN items, which tapped hyperactivity given that the SWAN includes items that tap two theoretically distinct subscales; with reference to the modification indices, we also iteratively added covariances within the SWAN inattentive items to secure good model fit. The 18 SWAN items have been found to measure a common latent trait as well as orthogonal factors or dimensions of inattention and hyperactivity/impulsivity (Normand, Flora, Toplak, & Tannock, 2012; Toplak et al., 2009). Thus, the SWAN latent variable here reflects this general factor and accounts for covariation among all the items on the SWAN (a two‐factor model of the SWAN items demonstrated that the inattention and hyperactivity latent variables are highly correlated (*r* = .86) suggesting commonality between these factors, see Figure S5). The proposed model in Figure 3 fit the data very well: CFI = .99, TLI = .99, RMSEA = .02, SRMR = .05.

Figure 3 shows that there is a strong concurrent relation between SOP and language (*r* = .56, *SE *= .10, 95% CIs [.37, .75], *p* < .001). SOP is also strongly related to SWAN ratings (*r* = .54, *SE* = .10, 95% CIs [.35, .73], *p* < .001).

A moderation analysis with latent variables was conducted in Mplus to investigate whether individual differences in inattention/hyperactivity as measured by the SWAN moderated the relation between SOP and language. After controlling for the main effects of SOP (*b* = .37, 95% CIs [.12, .63], *p* < .01) and SWAN ratings (*b* = .30, 95% CIs [.08, .52], *p* < .01), the SOP × SWAN interaction was significant (*b* = −.19, 95% CIs [−.33, −.04], *p* < .05); the overall pattern of results did not change when children with known diagnoses were excluded (see Figure S3), when the moderation was repeated using data from second assessment at age 7–8 (see Figure S4), when just inattention or hyperactivity factors from the two‐factor model were included as the moderator (see Figure S6), or when parent rather than teacher SWAN ratings were used (see Figures S7 and S8). Thus, the relation between SOP and language was moderated by children's level of inattention/hyperactivity as rated on the SWAN. This model explained 40% of the variance in children's language skills (*R* ^2^ = .40, *SE* = .07, *p* < .001). In short, the relation between SOP and children's language skills is affected by individual differences in children's level of inattentive/hyperactive behavior as rated on the SWAN.

**Figure 4:**
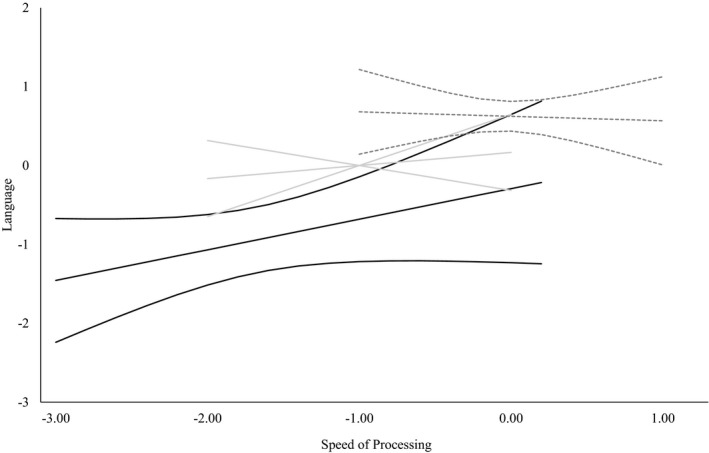
Simple slopes and 95% CIs for language and speed of processing at three levels of the inattention/hyperactivity latent variable: high Strengths and Weaknesses of Attention‐Deficit/Hyperactivity Disorder Symptoms and Normal Behavior scale (SWAN) scores (+1 *SD*), that is, fewer symptoms of inattention/hyperactivity (dark grey dash); medium SWAN scores, that is sample mean (light gray); and low SWAN scores (−1 *SD*), that is, more symptoms of inattention/hyperactivity (black).

To further understand the significant Inattention/Hyperactivity × SOP interaction we computed simple slopes for three bands of ratings: +1 *SD* (high SWAN scores reflective of good attention/behavior), 0 (sample mean SWAN score), and −1 *SD* (low SWAN scores reflective of greater inattention/hyperactivity). Figure 4 shows these three simple slopes with 95% CIs. The regression lines differ in both intercepts and slopes although they all intersect when SOP is about 1.2. The resulting coefficients are: .22 (95% CIs [−.19, .62]), .44 (95% CIs [.12, .75]), and .65 (95% CIs [.35, .95]) for high, average, and low SWAN scores, respectively. These indicate that when a child has above average scores on the SWAN (i.e., few symptoms of inattention/hyperactivity [dark gray]), the relation between SOP and language skills is weak (and not significantly different from zero), whereas when a child has below average scores on the SWAN (i.e., more symptoms of inattention/hyperactivity [black]), SOP is more strongly related to language.

### Does SOP Predict Children's Later Inattention/Hyperactivity?

Latent factors representing the continuously distributed symptoms dimensions underlying the diagnostic category of DLD and individual differences in inattention/hyperactivity, as well as a measure comprising indices of both verbal and nonverbal SOP were created using data from children who had SWAN questionnaires returned at both assessment points (*N* = 362, weighted to give estimates for the population). Our proposed measurement models fit the data well: CFI = .97, TLI = .97, RMSEA = .03 (90% CIs [.02, .04]), SRMR = .06 and CFI = .94, TLI = .93, RMSEA = .05 (90% CIs [.05, .06]), SRMR = .05, respectively.

**Figure 5:**
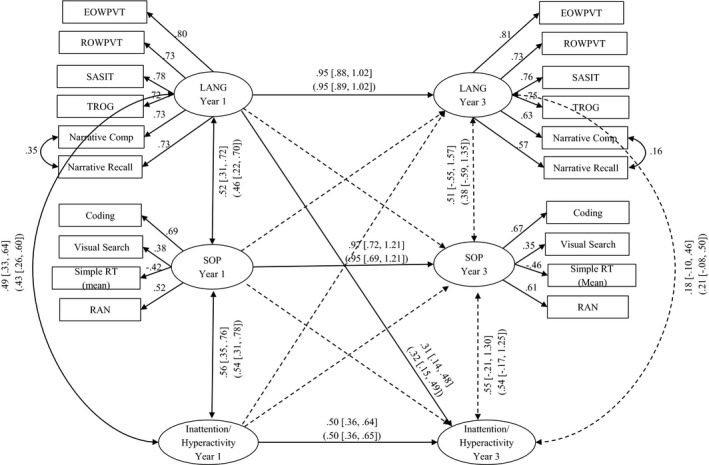
Longitudinal autoregressive path model with cross‐lagged effect showing the relation between speed of processing, language, and inattention/hyperactivity. Key standardized path estimates and correlation coefficients are depicted by single and double‐headed arrows, respectively (see Table S5 for standardized path estimates for the inattention/hyperactivity latent variable that range between .71–.94 and .65–.94 for Years 1 and 3, respectively). Path weights and confidence intervals for the whole sample are shown outside the brackets (*N* = 362), those for the sample without children with known diagnoses are shown inside the brackets (*N *= 318). Paths significant at the .05 level are represented by solid lines; nonsignificant paths by dashed lines. EOWPVT = Expressive One Word Picture Vocabulary Test; ROWPVT = Receptive One Word Picture Vocabulary Test; SASIT = School‐Age Sentence Imitation Test; TROG = Test for Reception of Grammar; RT = reaction time; RAN = rapid automatized naming.

To assess how SOP, language, and inattention/hyperactivity are related to each other longitudinally, a latent variable autoregressive path model with cross‐lagged effects was fitted to the data at age 7–8 (*N* = 362; see Figure 5). This model has overlap with that presented in Figure 2; however, here the longitudinal stability of inattention/hyperactivity (18 SWAN items) is assessed alongside that of SOP (coding, visual search, RAN, and mean RT) and language skills (receptive and expressive vocabulary, grammar, and narrative). In addition, this model assesses whether SOP and language predict additional variance in inattention/hyperactivity across time, once the autoregressive effect is controlled. If such longitudinal cross loadings were present, they would be consistent with (but not prove) a causal influence from the earlier to the later variable.

The resulting model, shown in Figure 5, gives a reasonable fit to the data: CFI = .92, TLI = .90, RMSEA = .05 (90% CI [.04, .05]), SRMR = .06. The figure shows standardized path weights together with their 95% confidence intervals for the whole sample (estimate for the sample excluding those with known medical diagnoses are shown in brackets). Paths significant at the .05 level are represented by solid lines, nonsignificant paths by dashed lines.

A number of features of this model are noteworthy. First, as demonstrated with the full sample on which language measures were available (Figure 2), the latent variables describing SOP and language remain highly stable. In comparison, the stability of the SWAN latent variable is a little weaker; this is likely due in part to completion of SWAN questionnaires by different teachers at the different assessment points but may also reflect the fact that inattention/hyperactivity are associated with some disorders in which these characteristics may change over time (Purper‐Ouakil, Wohl, Michel, Mouren, & Gorwood, 2004). The model accounted for 90% variance in SOP, 94% of the variance in language skills, and 58% of the variance in SWAN ratings at ages 7–8 years. Second, all cross lagged effects are small (≤ .08), and none are close to being significant except for the path between language at age 5–6 and SWAN ratings at age 7–8, which was significant (*b* = .31, *SE *= .09, 95% CIs [.14, .48], *p* < .001). This suggests that better early language skills are associated with better attention and behavior at ages 7–8 years.

## Discussion

This study tested the generalized slowing hypothesis of DLD (Kail, 1994), by evaluating the specific association between SOP and language in a large, epidemiological sample of school‐aged children from a range of socioeconomic backgrounds who were sampled from one of the more affluent counties in the United Kingdom. Given the association between poor SOP and other developmental disorders such as ADHD (Gooch et al., 2012; Kuntsi & Stevenson, 2000; Scheres et al., 2001), we considered whether any association between SOP and children's language skills varied according to children's levels of inattention/hyperactivity as rated by their teachers. Finally, we considered whether there was evidence to support the putative causal relation between weaknesses in SOP and DLD and/or symptoms of inattention/hyperactivity by asking whether individual variation in children's earlier (age 5–6) SOP predicted later (age 7–8) individual variation in children language skills and/or symptoms of inattention/hyperactivity.

We replicated previous findings that weaknesses in SOP are characteristic of children with DLD (Miller et al., 2001; Park et al., 2015); in our population sample, children with DLD performed more poorly than their TD peers on measures of SOP. The largest effect sizes were for measures of coding and visual search at age 5–6, with effects sizes diminishing over time. Children with DLD were also rated by their teachers as having more symptoms of inattention/hyperactivity compared to children without DLD at both assessment points.

The latent moderation analysis revealed a significant SOP × Inattention/Hyperactivity interaction in the prediction of children's language skill; thus, individual differences in inattention/hyperactivity moderated the relation between SOP and language. This effect was robust and evident at both testing points, when inattention and hyperactivity were considered separately, and when parent rather than teacher SWAN ratings were used (see Supporting Information). Simple slopes demonstrated that the relation between SOP and language was stronger for those with below average scores on the SWAN (i.e., with increased symptoms of inattention/hyperactivity) relative to those with above average scores on the SWAN (i.e., those with few symptoms of inattention/hyperactivity). This suggests that slow SOP may be particularly detrimental for language development in those children with more symptoms of inattention/hyperactivity and that good attention skills may protect against language difficulties in children with SOP deficits.

Our longitudinal findings indicate high stability of both language (Norbury et al., 2017) and SOP. Thus, there was no evidence, at least from school entry, that individual variation in earlier SOP predicted individual variation in later language skills (or vice‐versa) once autoregressive effects were controlled; an important step toward establishing causality. The stability of SWAN ratings was less strong, possibly reflecting differences between teachers at different testing points, developmental variation in attention control, or other contextual factors (Purper‐Ouakil et al., 2004). Nevertheless, despite a strong concurrent relation, there was again no evidence that individual variation in earlier SOP predicted individual variation in later inattention/hyperactivity or vice versa. Early language, however, was predictive of later ratings of inattention/hyperactivity. Although not the focus of this study, these findings are consistent with evidence of increased risk for ADHD over the course of development in children with DLD (Yew & O'Kearney, 2013).

The fact that weaknesses in SOP were more evident in those with higher levels of inattention/hyperactivity coupled with below average language skills (black line in Figure 5) is consistent with the idea that weaknesses in SOP may be a marker of comorbidity between DLD and ADHD, or a marker of developmental disorder more generally. Indeed, previous research supporting multiple deficit models suggest that SOP is a cognitive risk factor for ADHD and RD (McGrath et al., 2011; Shanahan et al., 2006), and that weaknesses in SOP reflect a domain general impairment that is common across a range developmental disorders (Bishop, 2013). This would also be consistent with recent research suggesting myelination supports processing speed in early childhood (Chevalier et al., 2015) and that reduced white matter density has been implicated in a number of neurodevelopmental disorders including DLD and ADHD (Fields, 2008). Thus, weaknesses in SOP could be viewed as a marker of neurodevelopmental disorder rather than a cause of any specific disorder such as DLD.

### Development and Stability

Age‐related increases in SOP have been associated with the development of more complex cognitive processes (e.g., reasoning) directly and indirectly (via effects on working memory; Kail, Lervåg, & Hulme, 2016). However, although there is some evidence that very early SOP (age 3 years) is directly related to later lexical skills (age 13; Rose et al., 2015), we did not observe this longitudinal link between SOP and language in our population sample during the first few years of school. It may be that this relation is only evident during an earlier period of development where nonverbal factors such as SOP play a critical role in establishing the foundations for later language learning. During the school years, language is a relatively stable construct with little change in the rank order of children over time (Bornstein, Hahn, & Putnick, 2016; Norbury et al., 2017); thus, there is little unexplained variance once autoregressive effects have been controlled.

### Limitations

It is important to note that the moderation and longitudinal analyses reported here cannot prove or disprove a “causal” relation among SOP, language, and inattention/hyperactivity. Although based on causal theory, at most the analyses reported here highlight concurrent associations between constructs. More robust evidence pertaining to the direction of any putative causal relation can be obtained from training studies. However, numerous studies indicate that training specific cognitive skills yields little generalization to other functional skills (Melby‐Lervag & Hulme, 2013). Thus, although there is some evidence to suggest that SOP is malleable (Mackey, Hill, Stone, & Bunge, 2011), training SOP alone would likely have negligible cascading impacts on language development. Research has also shown that psychostimulant treatment for ADHD has a positive effect on SOP (Kofler et al., 2013) and assessing whether these improvements have knock on effects on children's language skills would further elucidate the causal relations between SOP and language.

A second limitation of this work is that in the main analysis, individual differences in inattention/hyperactivity were assessed using only teacher SWAN ratings. This decision was made as more ratings scales were completed by teachers (65% and 74% at each respective testing point) than parents (57% and 54% at each respective testing point). Furthermore, there was less evidence of response bias in the teacher ratings (Hartman, Rhee, Willcutt, & Pennington, 2007). We conducted parallel moderation analyses using parent SWANs, which showed the same overall effect (see Supporting Information); however, future research should endeavor to obtain multiple and direct measures of children's symptoms of inattention/hyperactivity to improve reliability of measurement, although how to combine ratings from multiple sources is also a matter of debate (Bied, Biederman, & Faraone, 2017).

### Conclusions

In summary, our findings from a large‐scale, longitudinal population sample confirm that children with DLD perform more poorly then their TD peers on measures of SOP and have elevated symptoms of inattention/hyperactivity commonly associated with ADHD. This study is the first to demonstrate that symptoms of inattention/hyperactivity moderate the effect of SOP on language, but that early SOP does not predict later language, at least during the first few years of formal schooling. We therefore conclude that SOP is more likely a marker of comorbidity between DLD and developmental disorders associated with symptoms of inattention/hyperactivity (e.g., ADHD) or an indicator of neurodevelopmental disorder more generally.

## Supporting information


**Figure S1.** Recruitment Flow Diagram

**Figure S2.** Standard *z*‐Score Differences Between Children With and Without Developmental Language Disorder on Measures of Speed of Processing Excluding Children With Known Diagnoses (*N* = 61) in Year 1 (a) and Year 3 (b)

**Figure S3.** Path Model Showing the Effect of Inattention/Hyperactivity as a Moderator of the Relationship Between Speed of Processing and Language in Year 1 (95% CIs)

**Figure S4.** Path Model Showing the Effect of Inattention/Hyperactivity as a Moderator of the Relationship Between Speed of Processing and Language in Year 3 (95% CIs)

**Figure S5.** Measurement Model for the Continuously Distributed Dimensions of Speed of Processing, Language, Inattention, and Hyperactivity (as Measured by Teacher SWANs) in Year 1 (*N* = 343)

**Figure S6.** Path Model Showing the Effect of Inattention (Top) and Hyperactivity (Bottom; as Measured by Teacher SWANs) as Moderators of the Relationship Between Speed of Processing and Language (95% CIs)

**Figure S7.** Measurement Model for the Continuously Distributed Dimensions of Speed of Processing, Language, Inattention, and Hyperactivity (as Measured by Parent SWANs) in Year 1 (*N* = 299)

**Figure S8.** Path Model Showing the Effect of Inattention/Hyperactivity as Measured by Parent SWANs (*N* = 299) as a Moderator of the Relationship Between Speed of Processing and Language (95% CIs)

**Table S1.** Unweighted Frequencies of Children With Known Medical Diagnoses or Intellectual Impairment
**Table S2.** Psychometric Estimates for Measures
**Table S3.** Un weighted Sample Descriptives in Years 1 and 3
**Table S4.** Participant Characteristic for Those With Typically Developing Language and Developmental Language Disorder in Years 1 and 3 Excluding Children With Known Clinical Diagnoses (*N* = 61)**Table S5.** Standardised Path Estimates for the Inattention/Hyperactivity Latent Variable Depicted in Measurement Model in Figure 3 (*N* = 343) and in the Longitudinal Model Depicted in Figure 5 (*N* = 362)
