# Supplementary figures and images for: Does Inattention and Hyperactivity Moderate the Relation Between Speed of Processing and Language Skills?
Source: Child Dev. 2019 Feb 9;90(5):e565–83. doi: 10.1111/cdev.13220 (PMC6801354; doi:10.1111/cdev.13220)

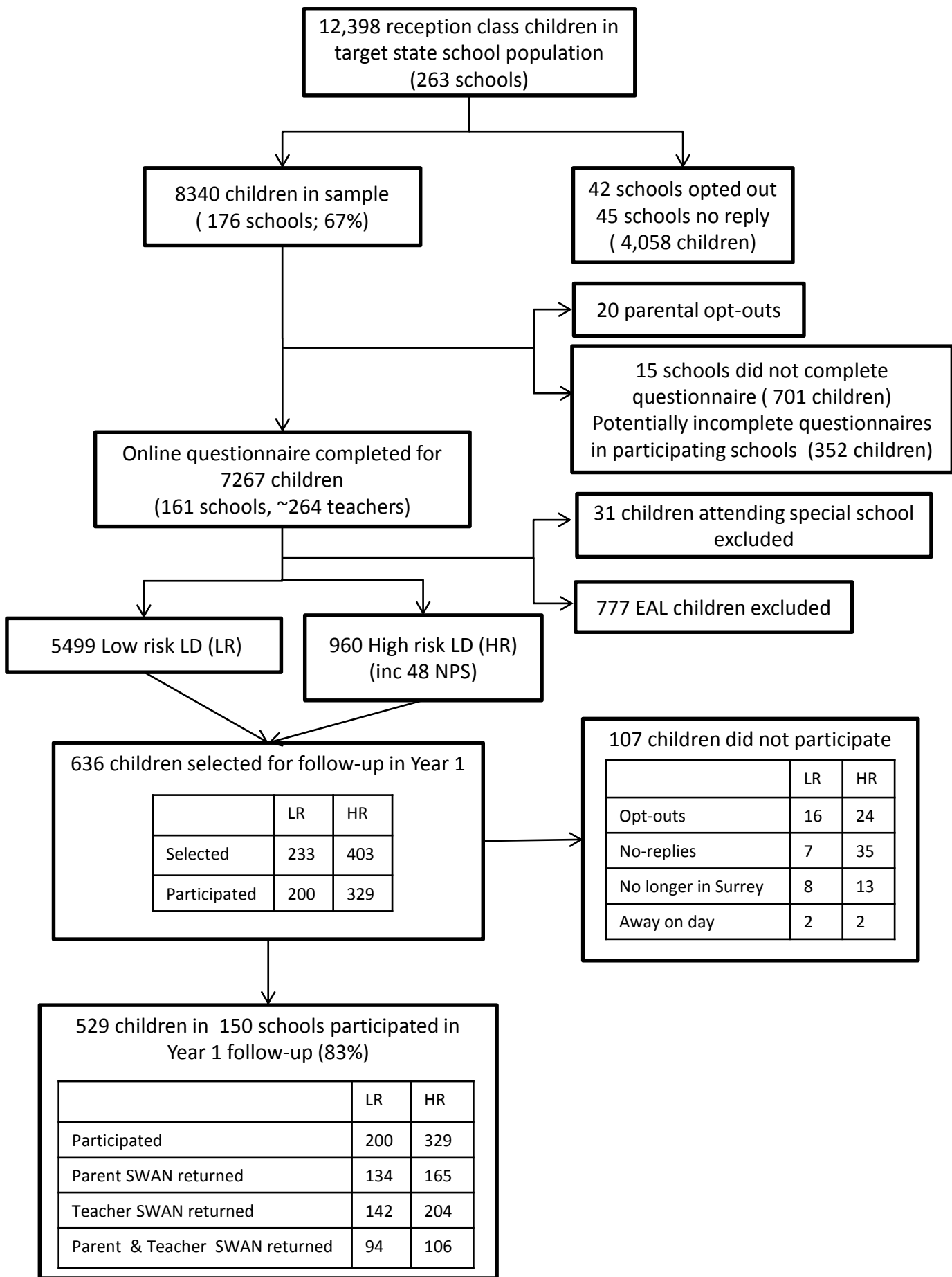

Supplement: Supplementary file 1 — Figure S1. Recruitment Flow Diagram [file CDEV-90-e565-s001.pdf]

(a)

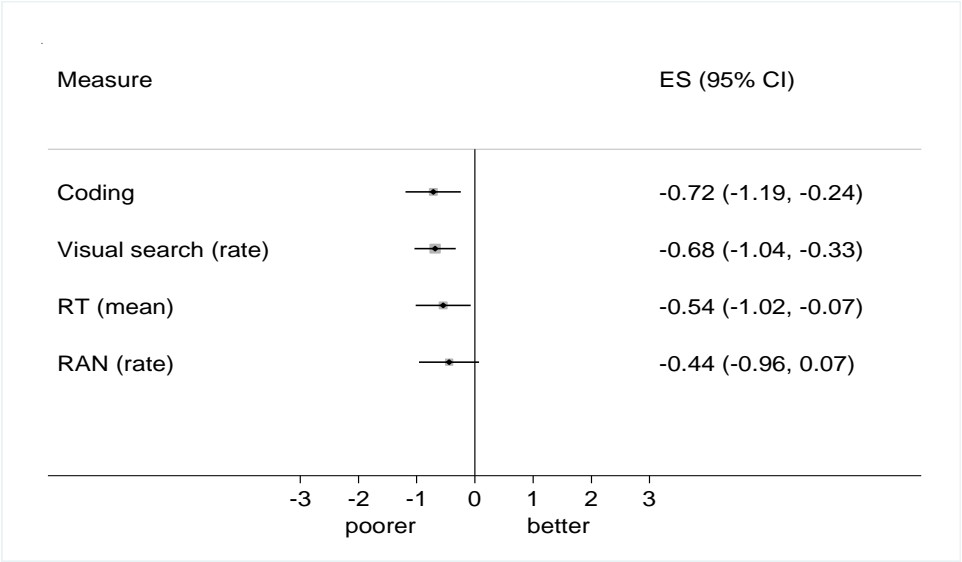

(b)

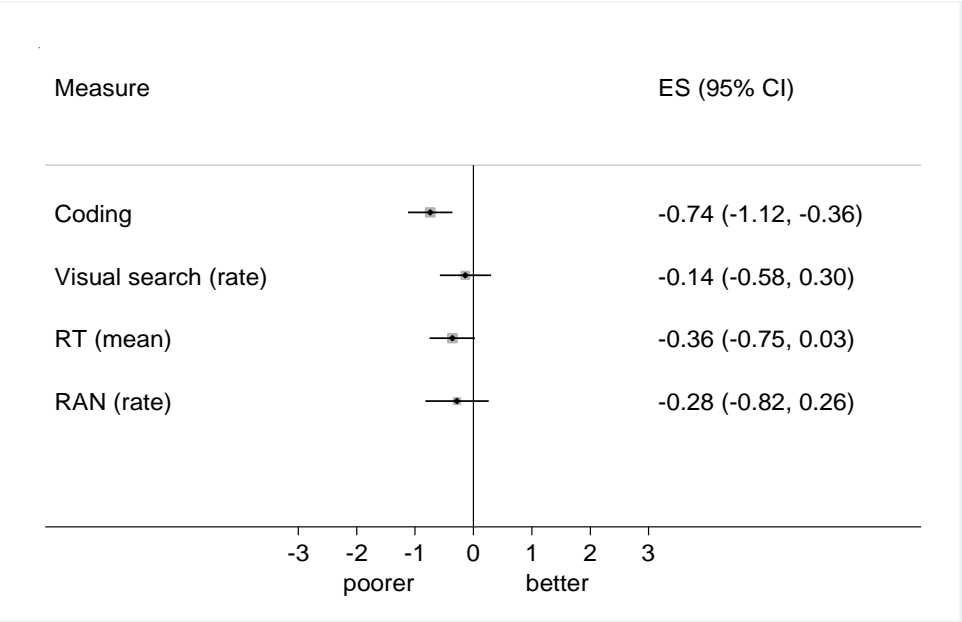

Supplement: Supplementary file 2 — Figure S2. Standard z‐Score Differences Between Children With and Without Developmental Language Disorder on Measures of Speed of Processing Excluding Children With Known Diagnoses (N = 61) in Year 1 (a) and Year 3 (b) [file CDEV-90-e565-s002.pdf]

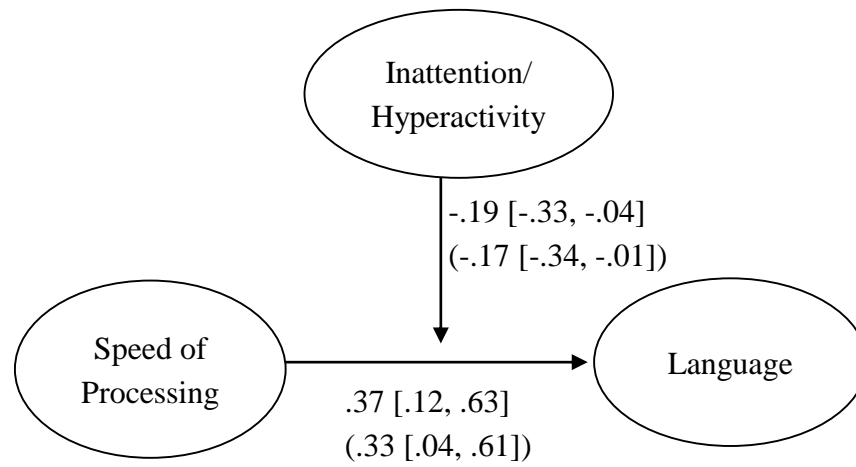

Supplement: Supplementary file 3 — Figure S3. Path Model Showing the Effect of Inattention/Hyperactivity as a Moderator of the Relationship Between Speed of Processing and Language in Year 1 (95% CIs) [file CDEV-90-e565-s003.pdf]

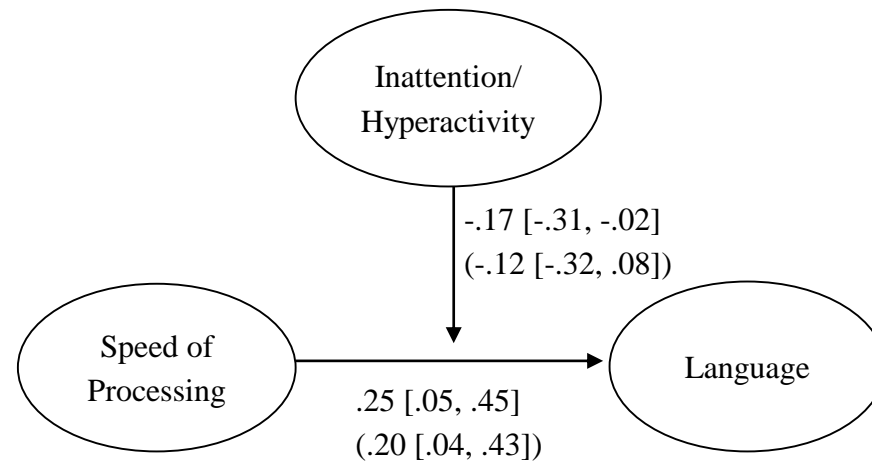

Supplement: Supplementary file 4 — Figure S4. Path Model Showing the Effect of Inattention/Hyperactivity as a Moderator of the Relationship Between Speed of Processing and Language in Year 3 (95% CIs) [file CDEV-90-e565-s004.pdf]

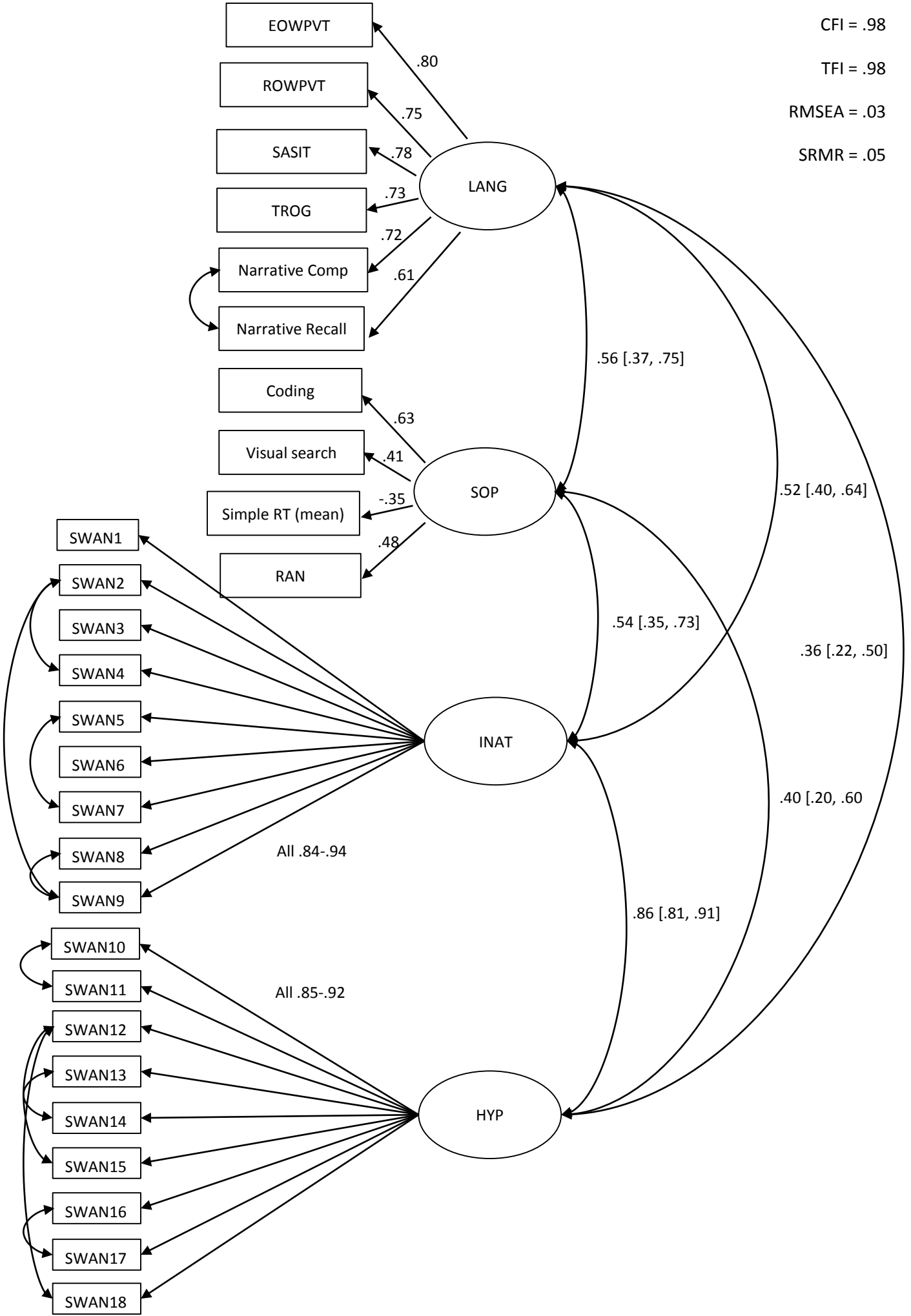

Supplement: Supplementary file 5 — Figure S5. Measurement Model for the Continuously Distributed Dimensions of Speed of Processing, Language, Inattention, and Hyperactivity (as Measured by Teacher SWANs) in Year 1 (N = 343) [file CDEV-90-e565-s005.pdf]

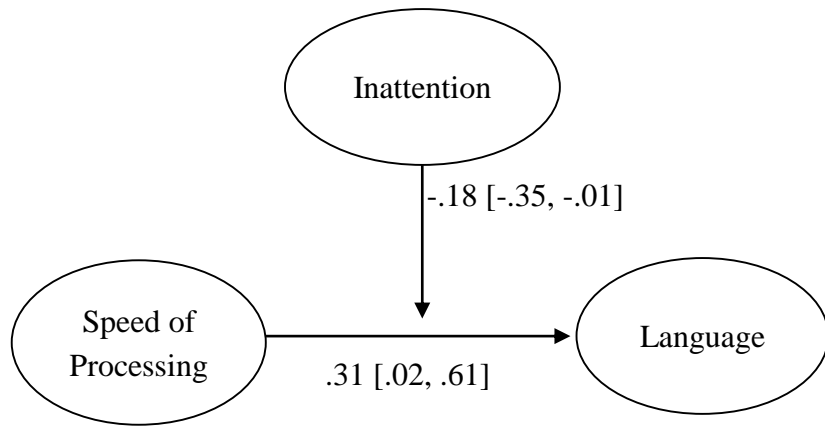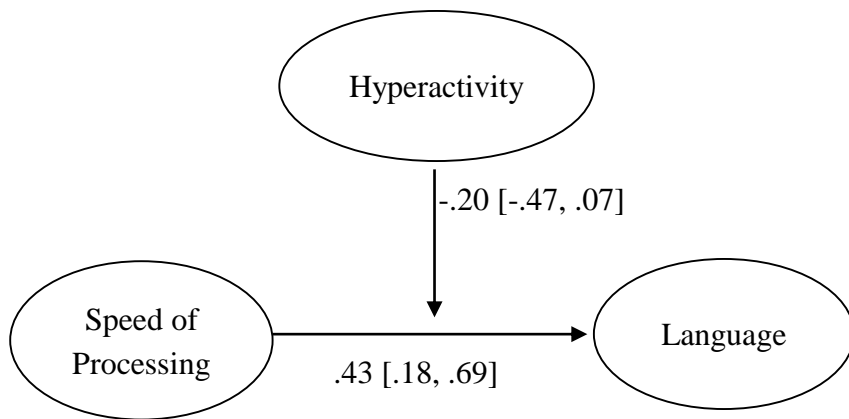

Supplement: Supplementary file 6 — Figure S6. Path Model Showing the Effect of Inattention (Top) and Hyperactivity (Bottom; as Measured by Teacher SWANs) as Moderators of the Relationship Between Speed of Processing and Language (95% CIs) [file CDEV-90-e565-s006.pdf]

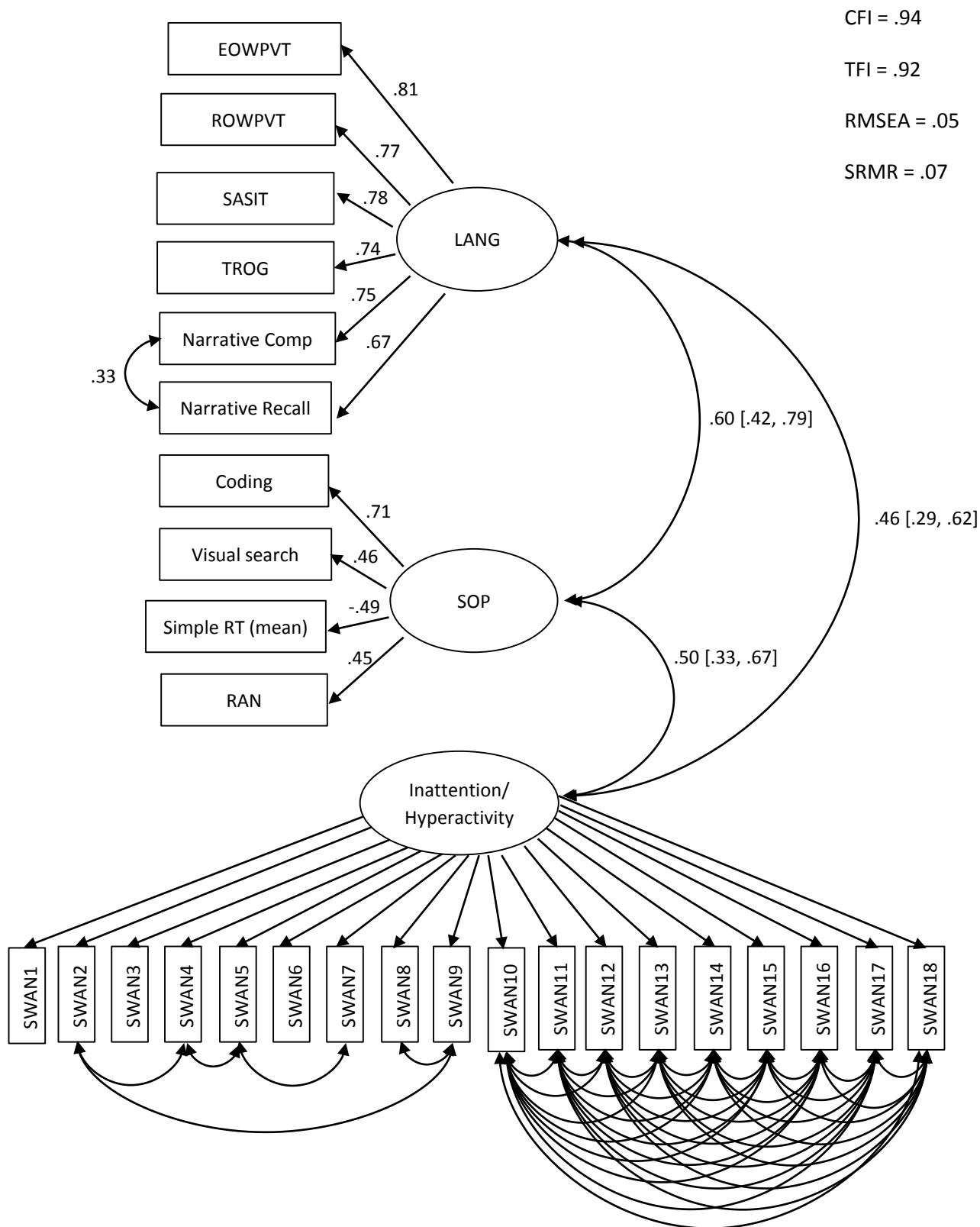

Supplement: Supplementary file 7 — Figure S7. Measurement Model for the Continuously Distributed Dimensions of Speed of Processing, Language, Inattention, and Hyperactivity (as Measured by Parent SWANs) in Year 1 (N = 299) [file CDEV-90-e565-s007.pdf]

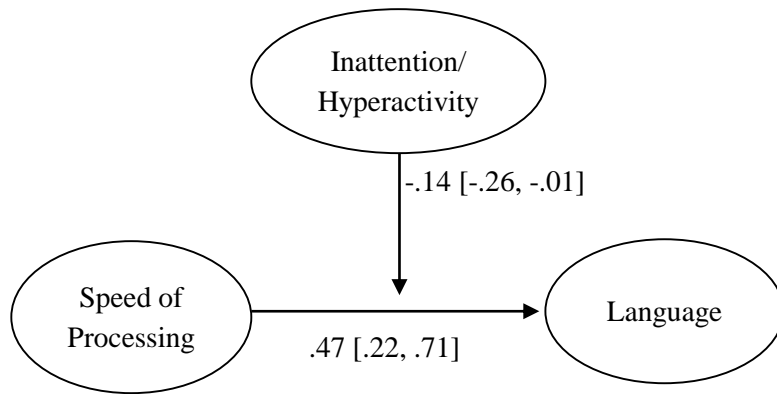

Supplement: Supplementary file 8 — Figure S8. Path Model Showing the Effect of Inattention/Hyperactivity as Measured by Parent SWANs (N = 299) as a Moderator of the Relationship Between Speed of Processing and Language (95% CIs) [file CDEV-90-e565-s008.pdf]
