# Supplementary material for: Does Inattention and Hyperactivity Moderate the Relation Between Speed of Processing and Language Skills?
Source: Child Dev. 2019 Feb 9;90(5):e565–83. doi: 10.1111/cdev.13220 (PMC6801354; doi:10.1111/cdev.13220)
Supplement: Supplementary file 9 — Table S1. Unweighted Frequencies of Children With Known Medical Diagnoses or Intellectual Impairment Table S2. Psychometric Estimates for Measures Table S3. Un weighted Sample Descriptives in Years 1 and 3 Table S4. Participant Characteristic for Those With Typically Developing Language and Developmental Language Disorder in Years 1 and 3 Excluding Children With Known Clinical Diagnoses (N = 61)Table S5. Standardised Path Estimates for the Inattention/Hyperactivity Latent Variable Depicted in Measurement Model in Figure 3 (N = 343) and in the Longitudinal Model Depicted in Figure 5 (N = 362) [file CDEV-90-e565-s009.docx]

Supplementary material

Figure S1 recruitment flow diagram


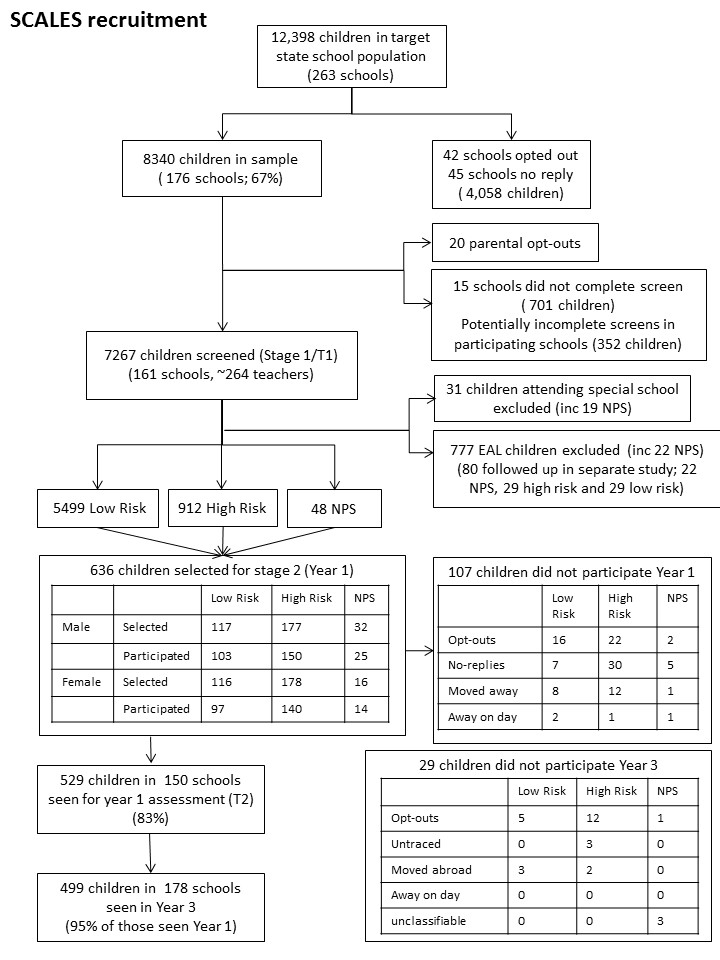


Core test battery administered at Stage 2

Non-verbal IQ

Each child completed Wechsler Preschool and Primary Scale of Intelligence *Block Design* and *Matrix Reasoning* subtests (WPPSI-III; Wechsler, 2003) according to the test manual.

*WISC-IV Block Design.* Each child was asked to copy patterns, presented to them in a stimulus booklet on the table, using red and white blocks. Trials were scored as correct or incorrect, time taken to complete pattern recorded and scored according to the manual. For items 1 – 3 the children were allowed two attempts and thus a score of 0 (incorrect), 1 (correct on trial 2), or 2 (correct on trial 1) was possible. Items 4 – 8 were scored as 0 (incorrect), or 4 (correct) and items 9 – 14 were scored according to time restrictions; children received more points for completely the item quickly. The task was discontinued after three consecutive scores of 0. Thus, a total raw score of 68 was possible. The average corrected stability coefficient is good (in the 0.80s).

*WISC-IV Matrix Reasoning.* Each item (maximum = 35) consists of an incomplete matrix with pictures or abstract patterns and designs. The child was required to select a picture, from a choice of five, that best completes the matrix. For each correct response the child was credited with one point. The task was discontinued after four incorrect responses on five consecutive items and possible scores range from 0 – 35. Matrix Reasoning has an excellent average test-retest reliability of r = .85 and an average internal consistency reliability of r = .89.

Core language battery

Children completed six tasks to assess receptive and expressive skills across multiple domains of language (vocabulary, grammar and narrative; Tomblin, Records, Buckwalter, Zhang, Smith, & O'Brien, 1997).

*Receptive One word Picture Vocabulary Test (ROWPVT-4; Martin & Brownell, 2000*). The child heard a word and had to select the corresponding picture, from a choice of four. This test taps semantic memory and draws less on phonological skills than measures of expressive vocabulary. The score on this task ranged from 0 to 190. Internal consistency for the age range assessed, 5- to 8-years, as rated by Cronbach’s Coefficient Alpha is 0.95 – 0.97. Test-retest reliability coefficients for raw scores is 0.97 and for standard scores is 0.91. This provides evidence that assessment stability of the same child across different time points is quite stable (Martin & Brownell, 2010).

*Expressive One Word Picture Vocabulary Test (EOWPVT; Martin & Brownell, 2000).* The child was asked to name objects, actions or concepts illustrated in pictures. The score on this task ranged from 0 to 190. Internal consistency for the age range assessed, 5- to 8-years, as rated by Cronbach’s Coefficient Alpha is 0.94 – 0.97. Test-retest reliability coefficients of raw and standard scores are 0.98 and 0.97 respectively (Martin & Brownell. 2011). Thus, the EOWPVT-4 is quite stable across time.

*Test of Reception of Grammar – Short Form (TROG-S).* The original test had 80 items which were condensed into 40. Children heard a sentence such as “the ball that is red is on the pencil” and were asked to select the corresponding picture out of a choice of four. If a child answered incorrectly on six consecutive items then the test was discontinued. Scores for this task range from 0 to 40, with a higher score indicating more correct responses. As noted in the manual, split-half reliability for the TROG-2 is 0.88 and the resulting correlation suggested good internal consistency (r = 0.877; Bishop, 2003).

*Sentence repetition (SASIT E32).* The child was asked to repeat 32 sentences out loud. All sentences were pre-recorded and played over headphones to the child one at a time, with a break for the child to repeat the sentence in between. Each repetition was audio-recorded and children were scored on whether the sentence was correct or incorrect (minimum score of 0, maximum score of 32), how many of the function words (range: 0-176) and content words (range: 0-121) were repeated correctly and whether the verb (range: 0-44) was correctly or incorrectly inflected. The child was given two practice trials to ensure that they understood the task.

*Assessment of Comprehension and Expression 6-11 (ACE 6-11; Adams et al., 2001).*

*Narrative Recall* The child was asked to listen to a story about a monkey in a forest. The story was pre-recorded and played over headphones with accompanying pictures displayed on a laptop computer. After listening to the story the child was asked to tell the story in their own words. The child was given a mark for each part of the story they correctly re-told. The child’s re-telling of the story was audio recorded and the score for this task ranged from 0 to 35. According to the manual, average internal consistency coefficient (Cronbach’s Alpha) of narrative propositions for children aged 6- to 11-years is 0.73.

*Narrative Comprehension*. Following the Narrative Recall task, the child was asked to answer 12 comprehension questions (6 literal and 6 inference questions) about the story they had just heard. The child was scored 0 for an incorrect answer, 1 point for a partially correct response and 2 points for a correct response. The partially correct and correct responses were determined by a written guide regarding the various possible responses. The score for this tasked ranged from 0 to 24.

| Table S1. Unweighted frequencies of children with known medical diagnoses or intellectual impairment. | | | |
| --- | --- | --- | --- |
| Primary diagnosis | LD | TD | Total |
| Hearing impairment | 0 | 3 | 3 |
| Visual Impairment | 1 | 1 | 2 |
| ASD | 14 | 6 | 20 |
| Epilepsy | 6 | 0 | 6 |
| Head injury/Neurological impairment | 2 | 0 | 2 |
| Cerebral Palsy | 1 | 0 | 1 |
| Down syndrome | 2 | 0 | 2 |
| Fragile X | 1 | 0 | 1 |
| Noonan syndrome | 1 | 0 | 1 |
| Neurofibromatosis | 1 | 0 | 1 |
| Intellectual Disability, no diagnosis (<=-2SD on tests) | 16 | 6 | 22 |
| Total | 45 | 16 | 61 |
|  |  |  |  |

Note: There were also children within the sample who were reported as having a diagnosis of ADHD (N=12: 3 DLD, 3 TD and 6 known diagnosis/intellectual impairment (4 DLD and 2 TD)), dyslexia (N=1: 1 TD) and developmental coordination disorder/motor difficulties (N=13: 6 TD, 3 DLD and 4 known diagnosis/intellectual impairment with DLD) by their parents/teachers. Given that these neurodevelopmental disorders are frequently comorbid with DLD in the literature but are not usually discussed as accounting for DLD per se these children were retained within the sample and not excluded from any analyses. Please note that the lower than expected prevalence of dyslexia is likely to be because this developmental disorder is not usually formally diagnosed until later in the school years. Here diagnoses are as reported by parents/teachers when the children were between 4-6 years old (T1/T2) and have not been verified by the research team or a clinical interview. Thus, any estimates should be treated with caution.

Table S2 Psychometric estimates for measures

|  | Test-retest reliability | Internal consistency (Cronbach’s alpha) | Inter-rater reliability |
| --- | --- | --- | --- |
| WISC-IV Block Design | .80 |  | - |
| WISC-IV Matrix reasoning | .85 | .89 | - |
| ROWPVT 4 | .97 | .95-97 | - |
| EOWPVT 4 | .98 | .94-.97 | - |
| TROG-S | - | .88 (split-half) | - |
| SASIT E32 | .98^e^ |  | - |
| ACE – Narrative Recall | .64 | 0.73 | - |
| ACE – Narrative Comprehension | - | - | - |
| SWAN (Total) | .76^a^; ICC = .92^b^ | .98 | ICC = .61, r = .64 (parent/teacher) |
| SWAN (Inattention) | .72^a^; ICC = .91^b^ | .98 | ICC = .60, r = .66 (parent/teacher) |
| SWAN (Hyperactivity) | .71^a^; ICC = .90^b^ | .97 | ICC = .51, r = .55 (parent/teacher) |
| RAN | .71^c^ | - | - |
| Visual Search | .59 (stability)^c^; .34^d^ | - | - |
| WPPSI-III Coding | .79 | - | - |
| Simple RT | .43 (stability)^d^ | - | - |
|  |  |  |  |

^a^ (Lakes, Swanson, & Riggs, 2012); ^b^ (Lai et al., 2013); ^c^ (Thompson et al., 2015); ^d^ Stability between SCALES T2 and T3; ^e^ (Chiat & Roy, 2013); - = unavailable

Table S3 Un-weighted sample descriptives in Years 1 and 3

|  | Year 1 | | | | | | Year 3 | | | | | |
| --- | --- | --- | --- | --- | --- | --- | --- | --- | --- | --- | --- | --- |
|  | N | Mean | SD | Min | Max | % missing | N | Mean | SD | Min | Max | % missing |
| Age | 528 | 71.63 | 4.72 | 61.00 | 82.00 | 0.00 | 499 | 95.23 | 4.52 | 85.00 | 111.00 | 0.00 |
| % Male | 528 | 52.46 |  |  |  |  | 499 | 51.7 |  |  |  |  |
| % White ethic origin | 528 | 90.15 |  |  |  |  | 499 | 89.78 |  |  |  |  |
| SES (IDACI rank) | 528 | 21366.39 | 7763.54 | 3908.00 | 32471.00 | 0.00 | - | - | - | - | - | - |
| NVIQ (z-score) | 528 | -0.43 | 1.09 | -4.85 | 2.78 | 0.00 | - | - | - | - | - | - |
| *Language measures (z-scores)* |  |  |  |  |  |  |  |  |  |  |  |  |
| EOWPVT | 528 | -0.48 | 1.05 | -3.12 | 2.40 | 0.00 | 499 | -0.52 | 1.15 | -3.84 | 4.38 | 0.00 |
| ROWPVT | 528 | -0.50 | 1.08 | -4.12 | 2.80 | 0.00 | 499 | -0.48 | 1.19 | -5.06 | 4.22 | 0.00 |
| Sentence Repetition | 528 | -0.63 | 1.21 | -3.04 | 1.84 | 0.00 | 499 | -0.54 | 1.32 | -5.55 | 1.49 | 0.00 |
| TROG-short | 528 | -0.51 | 1.08 | -2.76 | 2.01 | 0.00 | 499 | -0.44 | 1.14 | -2.76 | 2.16 | 0.00 |
| Narrative recall | 528 | -0.42 | 1.01 | -2.63 | 2.82 | 0.00 | 499 | -0.26 | 1.06 | -2.85 | 2.75 | 0.00 |
| Narrative comprehension | 528 | -0.50 | 1.13 | -2.96 | 2.53 | 0.00 | 499 | -0.39 | 1.15 | -2.89 | 2.68 | 0.00 |
| *Speed of processing measures* |  |  |  |  |  |  |  |  |  |  |  |  |
| Coding /65 | 508 | 31.80 | 12.33 | 3.00 | 64.00 | 3.79 | 483 | 45.89 | 11.91 | 5.00 | 65.00 | 3.21 |
| Visual search rate | 522 | 0.21 | 0.07 | 0.00 | 0.42 | 1.14 | 493 | 0.28 | 0.08 | 0.00 | 0.50 | 1.20 |
| RAN rate | 507 | 0.82 | 0.25 | 0.26 | 2.82 | 3.98 | 485 | 0.82 | 0.25 | 0.26 | 2.82 | 2.81 |
| SRT: mean RT (sec) | 511 | 492.78 | 89.62 | 290.30 | 887.94 | 3.22 | 489 | 419.26 | 91.22 | 262.59 | 1041.67 | 2.00 |
| *SWAN ratings (teachers)* |  |  |  |  |  |  |  |  |  |  |  |  |
| Inattention /63 | 343 | 33.21 | 13.03 | 9.00 | 63.00 | 35.04 | 362 | 34.30 | 13.22 | 9.00 | 63.00 | 27.45 |
| Hyperactivity /63 | 343 | 37.42 | 12.39 | 9.00 | 63.00 | 35.04 | 362 | 39.04 | 12.05 | 9.00 | 63.00 | 27.45 |
| Total /126 | 343 | 70.63 | 24.25 | 18.00 | 126.00 | 35.04 | 362 | 73.34 | 24.17 | 18.00 | 126.00 | 27.45 |

Table S4 Participant characteristic for those with typically developing language (TD) and Developmental Language Disorder (DLD) in Years 1 and 3 excluding children with known clinical diagnoses (N=61). Estimated means and frequencies reported, with 95% CIs in parentheses.

| Year 1 | TD | | DLD | | F (1, 466) | *p* |
| --- | --- | --- | --- | --- | --- | --- |
| N raw (estimated) | 376 | (5647) | 91 | (488) |  |  |
| % Male^a^ | 47.88 | (41.35, 54.48) | 54.26 | (3673, 70.79) | 0.44 | 0.51 |
| % White | 90.88 | (86.16, 94.10) | 92.54 | (86.18, 96.10) | 0.27 | 0.61 |
| Age | 71.73 | (71.09, 72.37) | 71.52 | (70.19, 72.85) | 0.08 | 0.78 |
| SES (IDACI rank) | 23896.1 | (22939.86, | 16243.01 | (13306.63, | 23.72 | <.001 |
|  |  | 24852.38) |  | 19179.38) |  |  |
| NVIQ (z-score) | 0.14 | (.02, .26) | -0.77 | (-.98, -.57) | 53.03 | <.001 |
| *Language measures (z-scores)* | |  |  |  |  |  |
| EOWPVT | 0.16 | (.04, .28) | -1.38 | (-1.56, -1.20) | 200.72 | <.001 |
| ROWPVT | 0.16 | (.04, .28) | -1.41 | (-1.70, -1.11) | 93.48 | <.001 |
| Sentence Repetition | 0.16 | (.05, .27) | -1.57 | (-1.85, -1.30) | 132.29 | <.001 |
| TROG-short | 0.14 | (.02, .26) | -1.15 | (-1.33, -.98) | 144.22 | <.001 |
| Narrative recall | 0.13 | (.01, .25) | -1.28 | (-1.58, -.98) | 71.92 | <.001 |
| Narrative comprehension | 0.17 | (.05, .28) | -1.35 | (-1.66, -1.03) | 78.6 | <.001 |
| *SWAN ratings* (teachers) |  |  |  |  |  |  |
| Inattention^b^ | 41.27 | (39.53, 43.01) | 30.01 | (24.12, 35.89) | 13.06 | <.001 |
| Hyperactivity^b^ | 43.48 | (41.72, 45.25) | 35.97 | (29.85, 42.09) | 5.38 | <.05 |
| Total^b^ | 84.75 | (81.40, 88.11) | 65.97 | (54.14, 77.81) | 9.03 | <.01 |
| Year 3 | TD | | DLD | | F (1, 440) | *p* |
| N raw (estimated) | 354 | (5633) | 87 | (498) |  |  |
| *Language measures (z-scores)* | |  |  |  |  |  |
| EOWPVT | 0.19 | (0.07, 0.31) | -1.34 | (-1.58, -1.10) | 124.02 | <.001 |
| ROWPVT | 0.17 | (0.04, 0.29) | -1.00 | (-1.27, -0.73) | 58.24 | <.001 |
| Sentence Repetition | 0.19 | (0.09, 0.29) | -1.35 | (-1.73, -0.98) | 60.48 | <.001 |
| TROG-short | 0.21 | (0.09, 0.32) | -1.18 | (-1.43, -0.93) | 97.87 | <.001 |
| Narrative recall | 0.18 | (0.06, 0.30) | -0.79 | (-1.19, -0.39) | 20.69 | <.001 |
| Narrative comprehension | 0.17 | (0.05, 0.29) | -1.06 | (-1.34, -0.78) | 63.96 | <.001 |
| *SWAN ratings (teachers)* |  |  |  |  |  |  |
| Inattention^c^ | 42.56 | (40.86, 44.27) | 27.84 | (22.93, 32.74) | 31.12 | <.001 |
| Hyperactivity^c^ | 44.32 | (42.63, 46.01) | 34.05 | ( 30.72, 37.38) | 29.29 | <.001 |
| Total^c^ | 86.88 | (83.63, 90.13) | 61.88 | (54.05, 69.72) | 33.61 | <.001 |

Note: ^a^ F statistic is a designed based corrected χ^2^ value; ^b^ Year 1: N = 302 (6,080.54); ^c^, Year 3: N=318 (6049.55); IDACI, Income Deprivation Affecting Children Index

(a)

(b)

Figure S2 Standard z-score differences between children with and without DLD on measures of speed of processing excluding children with known diagnoses (N=61) in Year 1 (a) and Year 3 (b). Error bars are 95% confidence intervals. Bars that cross the zero midline indicate no significant group difference. Boxes to the left of the zero indicate poorer performance in the DLD group.

Excluding those with known diagnoses (N=61), children with DLD performed significantly worse than children without DLD on 3/4 of the measures of speed of processing administered in Year 1 (see Figure S2): coding *F*(1, 461) = 8.81, *p*<.01, visual search, *F*(1, 465) = 14.51, *p*<.001 and simple RT, *F*(1, 463) = 5.15, *p*<.05. By Year 3 differences between children with and without DLD on measures of speed of processing were attenuated and the groups only differed significantly on the coding task, *F*(1, 437) = 14.64, *p*<.001).

-.19 [-.33, -.04]

(-.17 [-.34, -.01])

.37 [.12, .63]

(.33 [.04, .61])

Figure S3 Path model showing the effect of inattention/hyperactivity as a moderator of the relationship between speed of processing and language in Year 1(95% CIs). Path weights and confidence intervals for the whole sample are shown outside the brackets (N=343), those for the sample without children with known diagnoses are shown inside the brackets (N=302).

-.17 [-.31, -.02]

(-.12 [-.32, .08])

.25 [.05, .45]

(.20 [.04, .43])

Figure S4 Path model showing the effect of inattention/hyperactivity as a moderator of the relationship between speed of processing and language in Year 3 (95% CIs). Path weights and confidence intervals for the whole sample are shown outside the brackets (N=362), those for the sample without children with known diagnoses are shown inside the brackets (N=318).

Table S5 Standardised path estimates for the inattention/hyperactivity latent variable depicted in measurement model in Figure 3 (N=343) and in the longitudinal model depicted in Figure 6 (N=362).

| Inattention/Hyperactivity | Figure 3 | Figure 6 | |
| --- | --- | --- | --- |
|  |  | Year 1 | Year 3 |
| SWAN1 | 0.84 | 0.84 | 0.84 |
| SWAN2 | 0.93 | 0.93 | 0.93 |
| SWAN3 | 0.89 | 0.89 | 0.92 |
| SWAN4 | 0.92 | 0.91 | 0.94 |
| SWAN5 | 0.92 | 0.91 | 0.94 |
| SWAN6 | 0.94 | 0.94 | 0.94 |
| SWAN7 | 0.93 | 0.93 | 0.94 |
| SWAN8 | 0.90 | 0.89 | 0.82 |
| SWAN9 | 0.89 | 0.89 | 0.89 |
| SWAN10 | 0.82 | 0.83 | 0.77 |
| SWAN11 | 0.84 | 0.83 | 0.82 |
| SWAN12 | 0.80 | 0.78 | 0.80 |
| SWAN13 | 0.77 | 0.76 | 0.73 |
| SWAN14 | 0.80 | 0.80 | 0.80 |
| SWAN15 | 0.72 | 0.71 | 0.65 |
| SWAN16 | 0.75 | 0.76 | 0.75 |
| SWAN17 | 0.77 | 0.76 | 0.73 |
| SWAN18 | 0.78 | 0.77 | 0.79 |


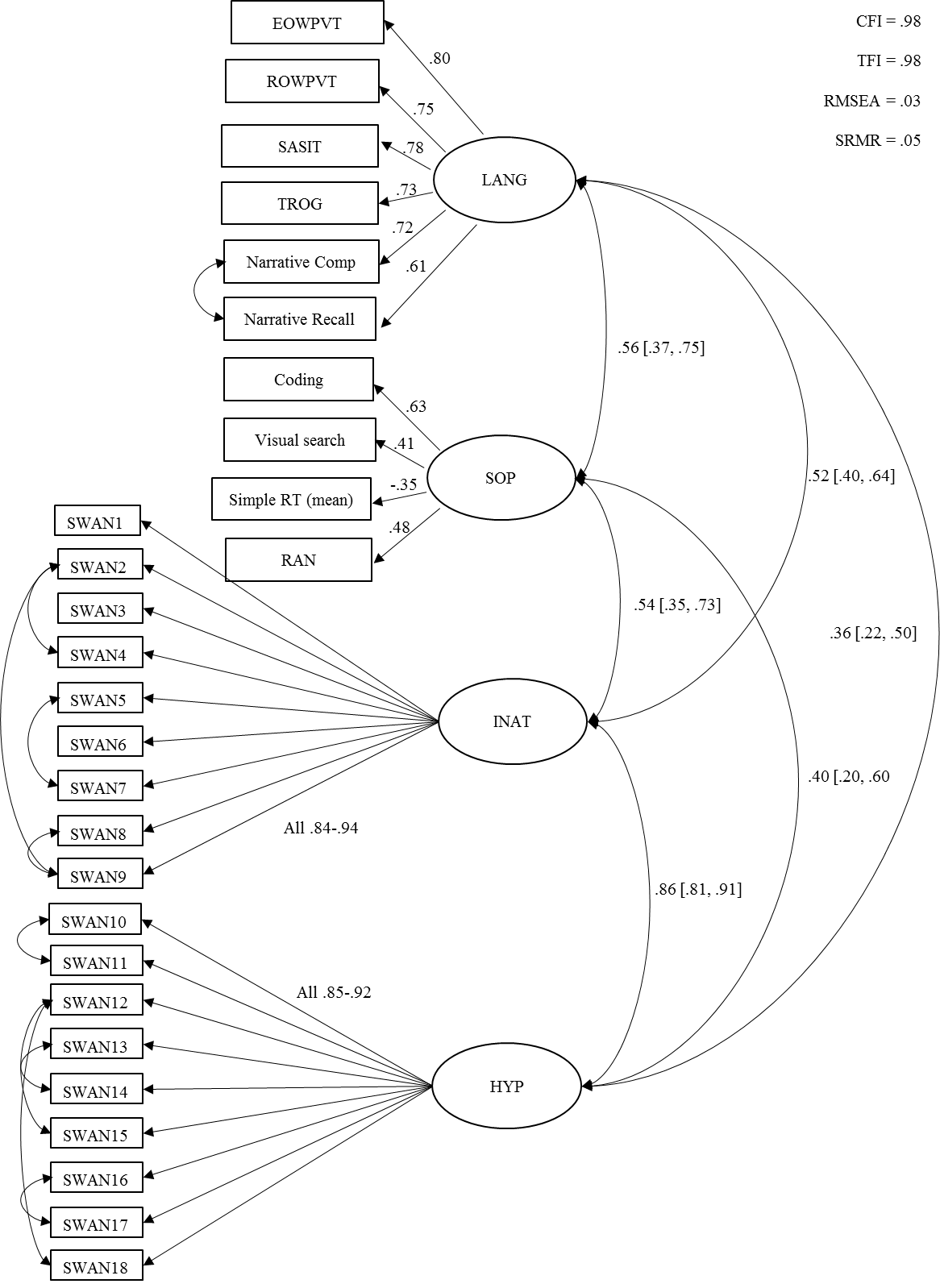


Figure S5 Measurement model for the continuously distributed dimensions of speed of processing, language, inattention and hyperactivity (as measured by teacher SWANs) in Year 1 (N=343). Standardised path estimates and correlation coefficients (with 95% CIs) are depicted by single and double-headed arrows, respectively.

Figure S6 Path model showing the effect of inattention (top) and hyperactivity (bottom) (as measured by teacher SWANs) as moderators of the relationship between speed of processing and language (95% CIs). Path weights and confidence intervals for the whole sample are shown (N=343).

-.20 [-.47, .07]

.43 [.18, .69]

-.18 [-.35, -.01]

.31 [.02, .61]


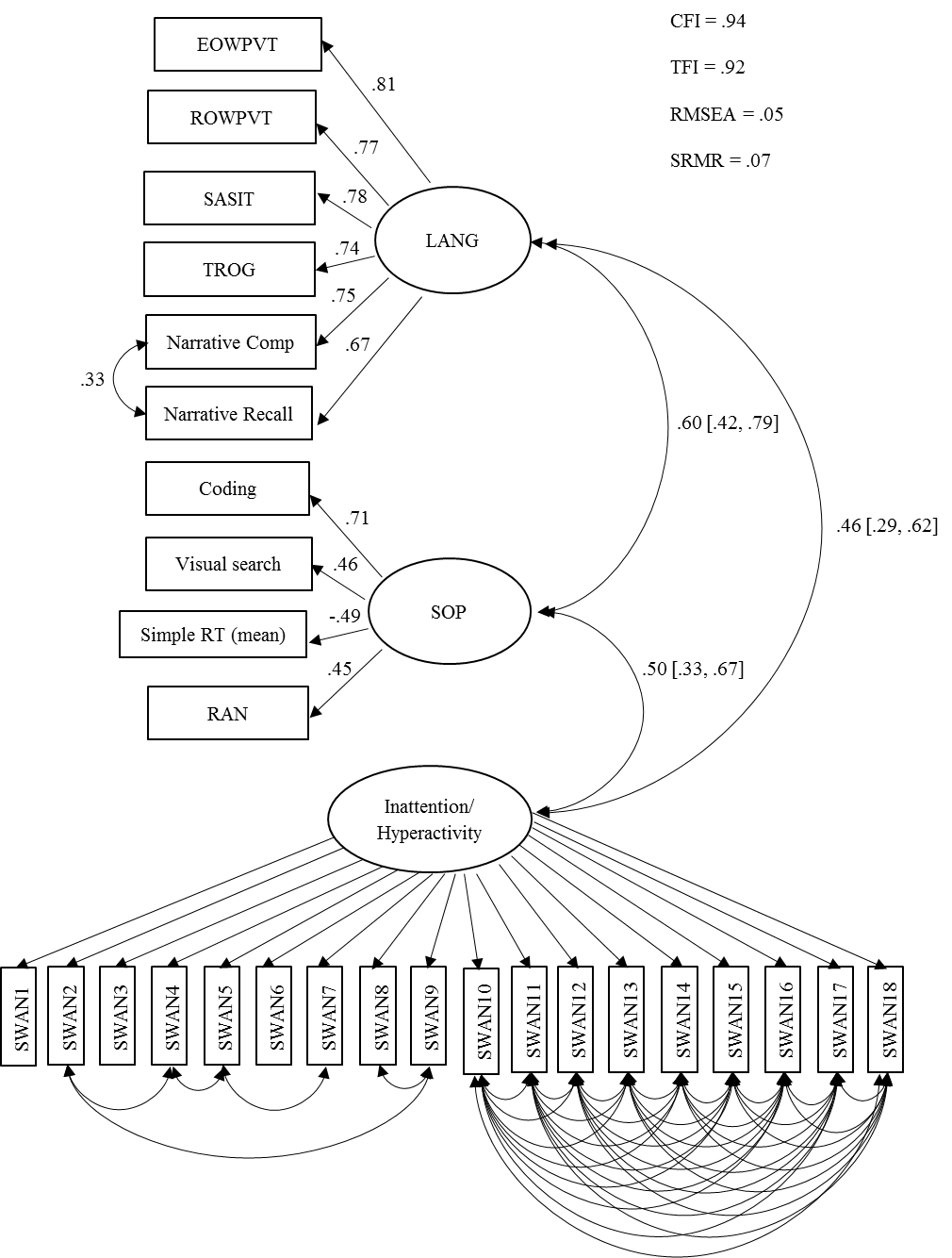


Figure S7 Measurement model for the continuously distributed dimensions of speed of processing, language, inattention and hyperactivity (as measured by parent SWANs) in Year 1 (N=299). Standardised path estimates and correlation coefficients (with 95% CIs) are depicted by single and double-headed arrows, respectively (all factor loadings for the inattention/hyperactivity latent variable range between .53-.84).

-.14 [-.26, -.01]

.47 [.22, .71]

Figure S8 Path model showing the effect of inattention/hyperactivity as measured by parent SWANs (N=299) as a moderator of the relationship between speed of processing and language (95% CIs).
